# Influence of obesity, parental history of diabetes, and genes in type 2 diabetes: A case-control study

**DOI:** 10.1038/s41598-019-39145-x

**Published:** 2019-02-26

**Authors:** Jaime Berumen, Lorena Orozco, Miguel Betancourt-Cravioto, Héctor Gallardo, Mirella Zulueta, Leire Mendizabal, Laureano Simon, Rosa Elba Benuto, Elisa Ramírez-Campos, Melissa Marin, Eligia Juárez, Humberto García-Ortiz, Angélica Martínez-Hernández, Carlos Venegas-Vega, Jesús Peralta-Romero, Miguel Cruz, Roberto Tapia-Conyer

**Affiliations:** 1 Unidad de Medicina Genómica, Hospital General de México, México City, Mexico; 2 Departamento de Medicina Experimental, Facultad de Medicina, Universidad Nacional Autónoma de México, Mexico City, Mexico; 3 Instituto Nacional de Medicina Genómica, Mexico City, Mexico; 4 Fundación Carlos Slim, México City, Mexico; 5 Patia Biopharma, Mexico City, Mexico; 6 Huella Génica, Mexico City, Mexico; 7 Servicio de Genética, Hospital General de México, Mexico City, Mexico; 8 Unidad de Investigación Médica en Bioquímica Hospital de Especialidades, Centro Médico Nacional Siglo XXI, Instituto Méxicano del Seguro Social, Mexico City, Mexico

## Abstract

Obesity, parental history (PH) of type 2 diabetes (T2D), and genes play an important role in T2D development. However, the influence of each factor on T2D variability is unclear. This study aimed to investigate the influence of obesity (body mass index [BMI], waist/hip ratio), PH, and 16 single-nucleotide polymorphisms (SNPs) associated with T2D on T2D variability in Mexico, comparing 1234 non-diabetic controls and 1219 diabetic patients. To replicate the data, a case-control (n = 2904) and a cross-sectional (n = 1901) study were also included. In a multivariate logistic regression model, all factors accounted for only 27.3% of T2D variability: SNPs (8.4%); PH (11.8%) and obesity (7.1%). These factors contributed more in men (33.2%) than in women (25%), specifically when the disease was diagnosed before the age of 46 (46.7% vs. 30%). Genes played a substantially more important role in men than in women (14.9% vs. 5.5%), while obesity and PH played a similar role in both genders. Genes and PH appeared to play a greater role than obesity in T2D. However, obesity contribution was calculated at the time of recruitment and may be underestimated in patients because the BMI decreased linearly with the number of years with the disease. The data suggest that sexual hormones may play important roles in genes that are associated with T2D.

## Introduction

The aetiology of type 2 diabetes (T2D) includes factors such as genes, genetic predisposition, ethnicity, poor nutrition, sedentary lifestyle, obesity, and dyslipidaemia^1^. Several family-based studies of disease heritability have indicated that T2D is strongly heritable^2–4^, and the heritability is on average 25%^5^. However, insufficient information exists on the heritability of T2D in non-twin families, and little is known regarding how much of this heritability is due to genes and other heritable factors, such as epigenetic factors^6^. Historical studies of linkage, candidate genes, and genome-wide association studies (GWAS) have discovered more than 100 variants of genes associated with T2D^7,8^. However, the influence of these genes on the disease is unclear. Based on their low individual odds ratios (ORs), most genes have very little influence on the development of the disease^8^. According to the results of a European case-control study, only approximately 10% of the T2D variability can be explained by T2D-susceptible loci^9–11^. Obesity is a modifiable factor that is clearly associated with the development of the disease. It is well known that the risk of T2D increases linearly as the body mass index (BMI) increases^12^. In fact, obesity has been promoted as the main risk factor for diabetes^13^. However, the relationship between T2D and obesity may not necessarily be as direct as it appears. For instance, in countries such as China, India, and Japan, in which the prevalence of T2D is high, the prevalence of obesity is relatively low^12,14^. In contrast, in countries such as Australia and the United Kingdom, where the obesity prevalence is high, T2D prevalence is relatively low^15,16^. In addition, although approximately two-thirds of people with diabetes are overweight or obese, only 2–13% of people who are obese develop T2D^13^. The percentage of T2D variability that is attributed to obesity has been poorly studied^17,18^.

Mexico is experiencing the most rapid increase ever recorded in the number of childhood and adult T2D cases^19^, and it now ranks second in Latin America and sixth in the world for T2D prevalence, with nearly 11.5 million affected patients^20^. The prevalence of T2D in Mexico (~18.9% [diagnosed plus undiagnosed])^21^ is more than twice that of populations of European origin (6.8%)^16^. Diabetes has been the leading cause of death in Mexico since 2000^21^, accounting for 15% of total mortality cases^22^. Shifts in dietary and physical activity patterns combined with genes that are highly associated with T2D may be contributing to this rise in prevalence. An extensive analysis of genetically susceptible loci in Mexican and Latin American individuals was recently performed by the Slim Initiative in Genomic Medicine for the Americas (SIGMA) GWAS study^23,24^. In this study, it was discovered that a deleterious variant of the gene *SLC16A11* is common in people of Mexican and Latin American descent (allele frequency of ~30%) but is rare in other populations. This variant alone could account for approximately 20% of the increase seen in T2D cases in Mexico^23–25^. However, the prevalence of obesity has increased markedly in Mexico over the last decades^26^. The prevalence of overweight and obesity in Mexico is approximately 70%^27^, ranking second after the United States among The Organization for Economic Co-operation and Development (OECD) countries^15^. Therefore, obesity has been attributed to have a substantial role in the development of T2D^28^.

Notably, only a few studies worldwide have attempted to discern the contribution of each risk factor and their interactions to T2D variability^29^. The epidemiology of the disease in Mexico presents an opportunity to investigate the role of risk factors in T2D. The objective of this study was, therefore, to investigate the influence of obesity (BMI, waist and hip circumference, waist/hip ratio [WHR]), parental history (PH) of T2D, and 16 genes strongly associated with T2D, as well as the interactions between them, in T2D variability. In addition, we investigated whether these factors differ in their effect on men and women and if they contribute to the early presentation of T2D. We conducted a case-control study and used univariate (ULR) and multivariate (MLR) logistic regression models to calculate the risk (OR) conferred and the percent contribution (r^2^) of each factor in T2D variability in Mexico. A clinical replica case-control study and a cross-sectional study were also included.

## Results

### Participants and demographic characteristics

Of the 1250 controls and 1250 cases included in the main case-control study, 16 controls and 31 cases were excluded due to the poor quality of their DNA samples. After the exclusion, a total of 1219 cases and 1234 controls remained. All 2453 participants were included for SNP and BMI analyses; however, only 2043 were included in the analysis of the PH of T2D, and only 1723 were included in the waist and hip circumference and WHR analysis, as the data of some participants were missing. Therefore, a total of 1413 participants were included in the MLR models. The demographic characteristics of the T2D patients and non-diabetic controls are presented in Table 1. The group of participants was 61.2% female and 38.8% male. The mean age of non-diabetic controls (57.7 ± 10.9) and cases (55.8 ± 11.7) was similar at the time of enrolment. The mean age at the time of diagnosis with T2D was 46.6 years (SD = 10.9), and the number of years with the disease varied over a wide range (0–46 years, mean = 9.1 ± 8.5). At the time of study recruitment, most patients (89%) were taking medication for their T2D and only a small percentage of cases (17.7%) and controls (17.3%) identified as smokers. The clinical data of the replica case-control study are very similar to the main case-control study (Supplementary Table S1).

**Table 1:** Selected clinical characteristic of main case-control study (n = 2453).

Variable	Women (n = 1501)		Men (n = 952)		Both genders (n = 2453)	
	Control (n = 759)	Cases (n = 742)	Control (n = 475)	Cases (n = 477)	Control (n = 1234)	Cases (n = 1219)
**Continuous variables: means ± SD (n)**						
Age (years)	57.2 ± 10.5 (759)	55.9 ± 11.9^b^ (742)	58.6 ± 11.4 (475)	55.7 ± 11.4^e^ (477)	57.7 ± 10.9 (1234)	55.8 ± 11.7^e^ (1219)
BMI (kg/m^2^)	28.3 ± 5 (755)	29.6 ± 5.6^e^ (737)	26.9 ± 4 (469)	28.7 ± 4.9^e^ (472)	27.8 ± 4.7 (1224)	29.2 ± 5.4^e^ (1209)
Waist (cm)	93.4 ± 11.1 (567)	97.5 ± 11.9^e^ (600)	93.5 ± 10.4 (315)	98.7 ± 12.7^e^ (287)	93.4 ± 10.9 (882)	97.9 ± 12.2^e^ (887)
Hip (cm)	103.3 ± 11.1 (541)	106.2 ± 12.2^e^ (597)	98.9 ± 7.8 (303)	101.7 ± 10.8^e^ (283)	101.7 ± 10.3 (844)	104.8 ± 11.9^e^ (880)
WHR	0.91 ± 0.07 (541)	0.92 ± 0.07^e^ (596)	0.94 ± 0.06 (303)	0.97 ± 0.06^e^ (283)	0.92 ± 0.06 (844)	0.94 ± 0.07^e^ (879)
Age at diabetes diagnosis (years)		46.9 ± 10.7 (738)		46.1 ± 11.1 (475)		46.6 ± 10.9 (1213)
Years with the disease		8.9 ± 8.1 (738)		9.5 ± 9 (475)		9.1 ± 8.5 (1213)
Parental diabetes history: % (n)*						
None	70 (420)	41.2 (265)^e^	70.7 (263)	41.6 (178)^e^	70.3 (683)	41.4 (443)^e^
Father	13.5 (81)	29.7 (191)	14.5 (54)	31.1 (133)	13.9 (135)	30.3 (324)
Mother	21 (126)	45.7 (294)	19.4 (72)	43.9 (188)	20.4 (198)	45 (482)
Both parents	4.5 (27)	16.6 (107)	4.6 (17)	16.6 (71)	4.5 (44)	16.6 (178)
Total	100 (600)	100 (643)	100 (372)	100 (428)	100 (972)	100 (1071)
**Smoking: % (n)**						
No	89.8 (657)	90.5 (665)	71.3 (326)	69.6 (329)	82.7 (983)	82.3 (994)
Yes	10.2 (75)	9.5 (70)^a^	28.7 (131)	30.4 (144)^a^	17.3 (206)	17.7 (214)^a^
Total	100 (732)	100 (735)	100 (457)	100 (473)	100 (1189)	100 (1208)
**T2D treatment: % (n)**						
No		10.2 (73)		12.6 (57)		11.1 (130)
Yes		89.8 (643)		87.4 (397)^a^		88.9 (1040)
Total		100 (716)		100 (454)		100 (1170)

BMI, waist and hip circumferences shown are those measured at enrollment. For the differences of means between the groups the p-values were assessed using the *t* test. For the differences in the frequency distribution between the groups the p-values were assessed with the chi-square test.

The superscripts indicate the p-values as follows: ^a^p > 0.05, ^b^p < 0.05, ^c^p < 0.01, ^d^p < 0.001 and ^e^p < 0.0001. *The sum of the percent of Non^e^, Father and Mother less the percent of both parents gives 100%.

In the cross-sectional study, the prevalence of T2D was 5.4% (104 out of 1914). Most cases were diagnosed during recruitment (n = 92), and only 12 cases had been previously diagnosed. The mean age of all cases was 49.2 ± 11.4 (n = 104), which was very similar to the age of the newly diagnosed cases (49.5 ± 11.3, n = 92). The latter, which corresponds to the age at diabetes diagnosis, was 3 years greater than that in the main case-control study (46.6 ± 10.9) and 4 years greater than in the clinical replica case-control study (45.4 ± 7.4). On the other hand, the mean age of the total control group was 38.6 ± 12.2, which is much lower than the mean of the controls used for the comparisons in the case-control studies (57.7 ± 10.9 and 60.1 ± 7.6). Therefore, for the comparison with the cases, we used the controls who were ≥50 years of age, whose mean age (58.7 ± 7.9) was close to the mean age of the controls in the case-control studies (Supplementary Table S2).

### Role of obesity in T2D

**Figure 1:**
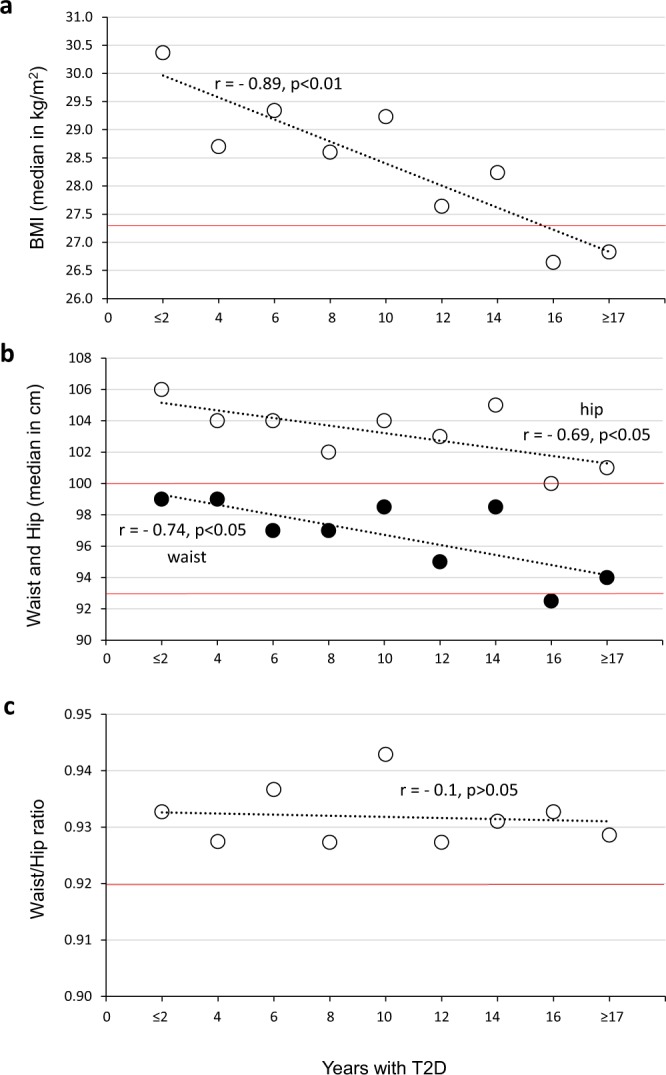
Trend of body mass index (BMI; panel a), circumferences of waist and hip (panel b) and WHR (panel c) over the years with type 2 diabetes. The red line indicates the median value in the control non-diabetic group. The correlation coefficient was calculated using the Pearson correlation test. BMI = body mass index, WHR = ratio of waist/hip circumference.

In the main case-control study, the mean BMI of cases (29.2 ± 5.4) was only somewhat higher than that of the controls (27.8 ± 4.7; Table 1). Although the difference in the means was statistically significant (p < 0.0001, *t* test) between the cases and controls, it appears too small in relation to the differences seen in other studies. Only a BMI >30 was associated with T2D (OR = 1.87, 95% CI 1.5–2.3, p < 0.001; Supplementary Table S3). Furthermore, in the ULR model, the measured BMI accounted for approximately only 2.8% (r^2^) of the variation in T2D in Mexico (Supplemental Table S3). This result contrasts with the important role attributed to obesity in the aetiology of T2D, and the discrepancy may be related to the number of years with the disease. Interestingly, the maximum peak in BMI was seen in patients who had ≤2 years with the disease (median = 30.4, IQR = 27.3–33.9), decreasing linearly to 26.8 (IQR = 23.7–30.2) in patients who had ≥17 years with the disease (r = −0.89, p < 0.01; Pearson correlation; Fig. 1a).

When we analysed the subset of T2D patients who had ≤2 years with the disease (n = 298) using ULR, the BMI accounted for approximately 9.6% (r^2^) of the T2D variability (Supplemental Table S4). It is remarkable that the r^2^ is higher in men (r^2^ = 17.4%) than in women (r^2^ = 6.6%). In particular, these figures contrast with the numbers observed in the group of patients with ≥3 years with the disease (r^2^ ≤ 1%; Supplemental Table S4). The data suggest that the numbers calculated for the group of patients who had the disease for ≤2 years should approach the BMI values of patients at the time of diagnosis. Therefore, to include all patients in the remaining analyses, we adjusted the BMI (adjBMI) of patients who had ≥3 years with the disease using the 100th-percentile of the BMI of the patients who had ≤2 years with the disease, as well as two equations derived from the data (see Methods and Materials).

The median adjBMI in the entire sample of patients (30.5, IQR = 27.3–33.9) was closer to the median raw BMI measured in the group of patients who had the disease for ≤2 years and accounted for approximately 12.6% (r^2^) of the T2D variability (Supplementary Table S3). Likewise, according to this result, the influence of BMI in T2D is higher in men (r^2^ = 18.1%) than in women (r^2^ = 10.3%) (Table 2). The analysis of the adjBMI nominal variable showed that the risks conferred by both pre-obesity and obesity for T2D were statistically significant and that the ORs increased up to 2.5-fold with the adjBMI compared to the values calculated with the raw BMI (Supplemental Table S3). Notably, the median raw BMI of cases (30.8, IQR = 26.9–34.1) and the value of r^2^ (0.138) observed in the cross-sectional study, including all cases (n = 104), were very close to those data obtained with the adjusted BMI in the main case-control study. However, if only the newly diagnosed cases (n = 92) are used in the analysis, the median (31.2, IQR = 27.3–34.2) and the r^2^ (0.149) are increased. Although the adjustment of the BMI that we made still seems to be below the true BMI that the cases would have when they were diagnosed, it is a closer mathematical approximation than the raw BMI measured during recruitment; therefore, we decided to use the adjusted BMI value for the calculations in the multivariate models of the main case-control study.

**Table 2:** Association of BMI, waist and hip circumferences and WHR with T2D stratified by sex using MLR models^a^.

Variable	Block	r^2^	p^b^	p^c^
**Women: controls (541)/cases (595)**				
BMI	1	0.084	<0.001	<0.001
Waist	2	0.091	0.009	<0.001
Hip	3	0.091	>0.05	<0.001
Waist/Hip ratio^d^	2	0.094	0.002	<0.001
**Men: controls (301)/cases (283)**				
BMI	1	0.157	<0.001	<0.001
Waist	2	0.157	>0.05	<0.001
Hip	3	0.157	>0.05	<0.001
Waist/Hip ratio^d^	2	0.168	0.023	<0.001
**Both genders: controls (842)/cases (878)**				
BMI	1	0.106	<0.001	<0.001
Waist	2	0.112	0.004	<0.001
Hip	3	0.114	0.048	<0.001
Waist/Hip ratio^d^	2	0.118	<0.001	<0.001

In cases, the BMI, Waist and Hip variables were adjusted with the years having T2D.

r^2^ = Nagelkerke method,

^a^The power (1-β err prob) >0.95 for the 3 multivariate logistic regression (MLR) models. ^b^Omnibus test (block), ^c^Omnibus test (model).

^d^The waist/hip ratio (WHR) was calculated with the raw waist and hip circumferences, measured when the cases and controls were recruited. It was introduced in the model in the block 2, instead of Waist and Hip variables introduced in blocks 2 and 3.

Like the BMI, the waist and hip circumferences also decreased linearly in diabetic patients as the number of years that they had the disease increased (Fig. 1b). Similarly, the value of r^2^ for both parameters is greater in patients who had T2D for ≤2 years than in those who had more than 2 years with the disease (Supplementary Table S4). In contrast, the WHR remained more or less constant, at approximately 0.93, and did not vary with the number of years with T2D (Fig. 1c). The waist and hip circumferences were adjusted using the same method as the BMI (see Materials and Methods). The median adjusted waist and hip circumferences of all patients were similar to those calculated with the raw values in patients that had ≤2 years with the disease (99, IQR = 92–105.4 and 105.7, IQR = 98.7–112.7, respectively). In the ULR models, it was clearly observed that the adjusted waist and hip circumference and the WHR do not contribute more than the adjusted BMI to the T2D variability (Supplementary Tables S3 and S5). In fact, in the MLR models, when the BMI is introduced in the first block, the additional contribution of the waist and hip circumference and the WHR in the explanation of the T2D are very small (Table 2). Of these three variables, the WHR, which was calculated with the raw waist and hip circumference values, showed the best performance in the MLR models.

**Figure 2:**
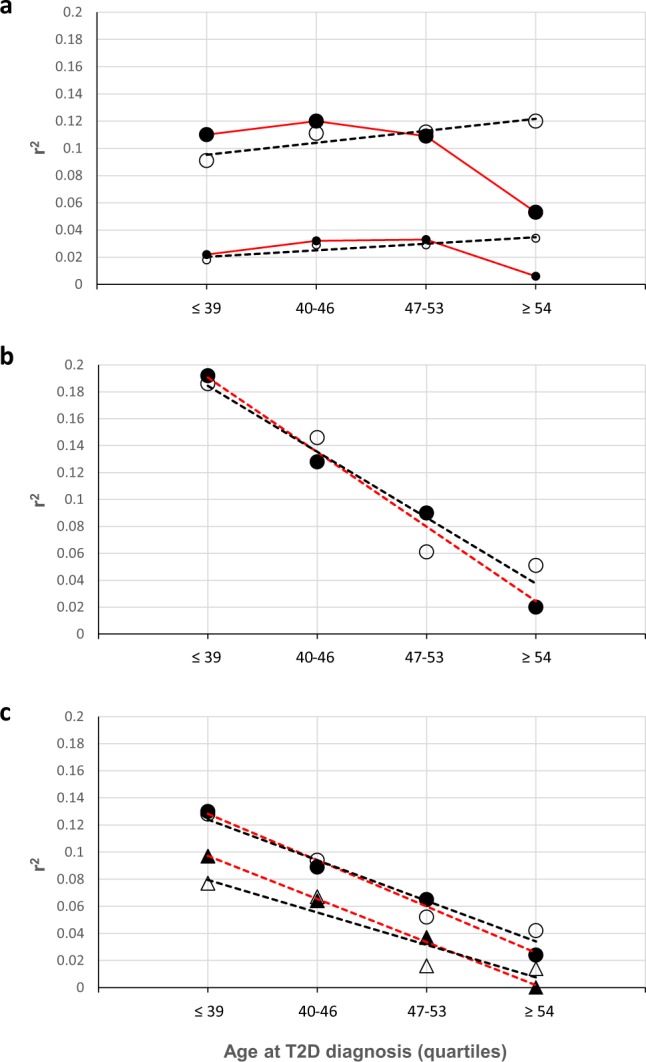
Trend of the influence (r^2^) of BMI (panel a) and parental history of T2D of both parents together (panel b) and of the mother and the father separately (panel c) in type 2 diabetes, according to age at T2D diagnosis, grouped by quartiles. The closed circles and triangles show the information of the main case-control study, and the open circles and triangles show the information of the clinical replica study. The dashed lines in red represent the regression lines of the main case-control study and those in black, the regression lines of the clinical replica study. In panel a, the large circles represent the adjusted BMI, and the small circles represent the raw BMI. In panel c, the circles represent the influence of the mother, and the triangles represent the influence of the father. All regressions were carried out with the Pearson’s correlation test and p-values < 0.01.

Similar figures were seen in the clinical replica. The median BMI decreased over the years of having T2D, similar to how it decreased in the cases of the main case-control study. The median of the waist and hip circumferences fell during the first 12 years of the disease, then rose, and decreased again, while the WHR remained more or less constant (Supplementary Fig, S1). The r^2^ values obtained with the raw BMI were very similar to those obtained in the main case-control study. The r^2^ obtained with the adjusted BMI was 0.077 (p < 0.001, omnibus test) and increased to 0.086 (p < 0.001, omnibus test) when the WHR was introduced into the model. Interestingly, the trend of the influence of BMI (r^2^) is slightly different between the two case-control studies as increased the age at T2D diagnosis (Fig. 2a). In the main study, the r^2^ remained more or less constant (~0.12) between the individuals diagnosed at age ≤39 to 53 years, and subsequently, the value of the r^2^ decreased to 0.053. In contrast, in the clinical replica, the r^2^ slightly increased from individuals diagnosed at ≤39 years (r^2^ = 0.091) to those diagnosed ≥54 years old (r^2^ = 0.12).

### Role of PH of T2D

The PH of T2D was different between cases and controls (p < 0.001, Pearson chi-squared; Table 3). A total of 58.6% of cases had a PH of T2D compared to only 29.3% of controls. The majority of controls (25.2%) and 42% of cases with a positive history had only one affected parent. Notably, 16.6% of cases had two affected parents, compared with only 4.5% of controls. Furthermore, the OR for two positive parents was considerably higher than the OR for only one positive parent (6.24 vs. 2.83, respectively; Table 3). According to the r^2^ of the ULR model, the role of PH in T2D in Mexico is approximately 12.1% across the entire population (Table 3). Upon analysing the separate influence of the father and the mother, the influence of the mother (r^2^ = 9% was found to be greater than the influence of the father (r^2^ = 5.2%). When we stratified the data by gender, no differences were found when comparing each gender with the entire sample. The figures were similar in the comparisons of the clinical replica. Although the value of r^2^ was lower in these comparisons, the influence of the mother was twice the influence of the father in having T2D (r^2^ = 0.052 vs. r^2^ = 0.026; Table 3).

**Table 3:** Association of parental history of T2D with T2D using ULR models^*^.

Parental history of T2D	Controls % (n)	Cases % (n)	Univariate Logistic Regression	
			r^2^	OR (95CI)
**Main case-control study**				
Father				
negative	86.1 (837)	69.7 (747)	0.052^e^	1
positive	13.9 (135)	30.3 (324)^e^		2.69^e^ (2.15–3.36)
Mother				
negative	79.6 (774)	55 (589)	0.09^e^	1
positive	20.4 (198)	45 (482)^e^		3.2^e^ (2.63–3.9)
Both parents				
0	70.3 (683)	41.4 (443)	0.121^e^	1
1	25.2 (245)	42 (450)		2.83^e^ (2.33–3.45)
2	4.5 (44)	16.6 (178)		6.24^e^ (4.39–8.86)
Total	100 (972)	100 (1071)^e^		
**Replica case-control study**				
Father				
negative	82.9 (344)	67.6 (1667)	0.026^e^	1
positive	17.1 (71)	32.4 (799)^e^		2.32 (1.77–3.04)^e^
Mother				
negative	76.4 (317)	52.7 (1306)	0.052^e^	1
positive	23.6 (98)	47.3 (1170)^e^		2.9 (2.28–3.68)^e^
Both parents				
0	63.4 (263)	36.8 (905)	0.073^e^	1
1	32.5 (135)	47.2 (1161)		2.5 (2–3.13)^e^
2	4.1 (17)	16 (394)^e^		6.74 (4.07–11.16)^e^
Total	100 (415)	100 (2460)		

r^2^ = Nagelkerke method.

*The power (1-β err prob) >0.95 for all univariate logistic regression (ULR) models.

p-value for frequency distribution was calculated with the Pearson chi-square test, for r^2^ with the Omnibus test, and for OR with the Wald test. The p-values are indicated with super indices as follows: ^a^p > 0.05, ^b^p < 0.05, ^c^p < 0.01, ^d^p < 0.001 and ^e^p < 0.0001.

In contrast, in the cross-sectional study, although the parental history was only explored as positive or negative, the association between PH and T2D was very low (r^2^ = 0.022). This could be related with the fact that those cases were diagnosed 3–4 years older than the cases of the case-control studies (45.4 or 46.6 vs. 49.5). In fact, when we stratified the cases of the case-control studies by the quartiles of age at diagnosis, a greater influence of PH, in both the main study and replica study, was observed in the first quartile (≤39 years; r^2^ = ~0.19). The value of r^2^ then decreased linearly to the last quartile (≥54 years; r^2^ = 0.02 or 0.05; Fig. 2b). Interestingly, in both case-control studies, the influence of the mother and father fell in parallel as the age at T2D diagnosis increased (Fig. 2, panel c).

### Role of 16 selected genes in T2D

The frequencies of the risk alleles (RAs) and the ORs of the 16 selected SNPs were similar to those previously reported for the Mexican population, but with interesting differences. For instance, the OR of the SNP from *SLC30A8* was >1, and the ORs of the SNPs from, *PPARG*, *IGF2BP2*, *ADCY5*, *KCNQ1*, *TCF7L2 and CDKN2B* were slightly higher than those reported previously (Supplementary Table S6). The observed frequencies of the genotypes of all SNPs in the control sample were similar to the frequencies expected based on the allelic frequency and the Hardy-Weinberg law (p > 0.05, chi-squared test). The frequencies of the genotypes of eight SNPs (from genes *SLC16A11, INS-IGF2, SLC30A8, IGF2BP2, ADCY5, KCNQ1, TCF7 L2, and FTO*) were different and statistically significant between the cases and controls (Supplementary Table S7). The adjusted ORs or r^2^ values of all but one of these SNPs (*FTO*) and the SNPs of *HNF1A* and *CDKN2B* genes were statistically significant at p < 0.1 in the MLR model (Supplementary Table S7 and Table 4). The total contribution of these nine SNPs to T2D in Mexico was 6.5% according to the MLR model. For at least seven genes (*SLC16A11, SLC30A8*, *IGF2BP2, ADCY5*, *KCNQ1, TCF7L2* and *CDKN2B*), the homozygous genotype with the RA conferred a higher risk than the heterozygous genotype (Table 4). These results suggested an additive effect for the risk of T2D for these SNPs.

**Table 4:** Association of nine SNPs with T2D in a sample of cases and controls from Mexico City stratified by gender (n = 2453)*.

Gene (SNP)	Number of Risk Alleles	Multivariate logistic regression model (controls/cases)					
		Men (475/477)		Women (759/742)		Both genders (1234/1219)	
		OR (95% CI)	p-value	OR (95% CI)	p-value	OR (95% CI)	p-value
SLC16A11 (rs75493593)	0	1	3.1E-02	1	4.2E-03	1	9E-05
	1	1.44 (1.1–1.9)	1.7E-02	1.44 (1.1–1.8)	2.1E-03	1.45 (1.2–1.7)	7.4E-05
	2	1.48 (1–2.2)	4.2E-02	1.44 (1.1–1.9)	1.4E-02	1.48 (1.2–1.9)	1E-03
INS-IGF2** (rs149483638)	0	1	4.0E-03	1	6.4E-02	1	1.2E-03
	1	2.41 (1.4–4.2)	1.9E-03	1.54 (1–2.3)	2.7E-02	1.76 (1.3–2.4)	4.2E-04
	2	2.48 (1.4–4.3)	1.1E-03	1.55 (1.1–2.3)	2.2E-02	1.75 (1.3–2.4)	4.1E-04
HNF1A (rs483353044)	0	1		1		1	
	1	2 (0.6–7)	>0.1	1.97 (0.7–5.9)	>0.1	1.89 (0.8–4.3)	9.8E-02
SLC30A8 (rs3802177)	0	1	9.2E-02	1	2.3E-02	1	4.7E-03
	1	1.04 (0.8–1.4)	>0.1	1.32 (1.1–1.6)	1.0E-02	1.22 (1–1.4)	2.3E-02
	2	1.77 (1.1–3)	3.1E-02	1.42 (0.9–2.2)	9.4E-02	1.61 (1.2–2.2)	3.6E-03
IGF2BP2** (rs4402960)	0	1	2.3E-02	1	>0.1	1	3.4E-02
	1	1.45 (1.1–2)	1.4E-02	1.11 (0.9–1.4)	>0.1	1.22 (1–1.5)	2.8E-02
	2	1.88 (0.8–4.3)	>0.1	1.25 (0.7–2.3)	>0.1	1.49 (0.9–2.4)	9.9E-02
ADCY5 (rs11717195)	0	1	>0.1	1	>0.1	1	8.8E-02
	1	0.92 (0.6–1.4)	>0.1	1.1 (0.8–1.5)	>0.1	1.04 (0.8–1.3)	>0.1
	2	1.13 (0.7–1.7)	>0.1	1.29 (0.9–1.8)	>0.1	1.25 (1–1.6)	8.3E-02
KCNQ1 (rs2237897)	0	1	2.2E-06	1	4.8E-04	1	5.3E-09
	1	1.66 (1.1–2.5)	1.6E-02	1.53 (1.1–2.1)	1.3E-02	1.53 (1.20–2)	1.3E-03
	2	2.74 (1.8–4.1)	2.0E-06	1.92 (1.4–2.7)	1.3E-04	2.17 (1.7–2.8)	5.4E-09
TCF7L2** (rs7903146)	0	1	3.4E-04	1	>0.1	1	4E-04
	1	1.89 (1.4–2.6)	1.0E-04	1.25 (1–1.6)	7.6E-02	1.47 (1.2–1.8)	9.4E-05
	2	2.11 (0.6–7.3)	>0.1	0.8 (0.3–2.4)	>0.1	1.51 (0.7–3.3)	>0.1
CDKN2B*** (rs10811661)	0	1	>0.1	1	6.5E-02	1	9.9E-02
	1	1.67 (0.2–12.9)	>0.1	2.42 (0.7–7.9)	>0.1	2.35 (0.9–6.5)	9.8E-02
	2	1.59 (0.2–12.1)	>0.1	3.01 (0.9–9.6)	6.2E-02	2.64 (1–7.1)	5.6E-02

p-values were calculated with the Wald test.

*The power (1-β err prob) >0.95 for all SNPs with a p < 0.1, except for HNF1A (rs483353044) and IGF2BP2 (rs4402960) which power value were 0.86 and 0.88, respectively, in both genders MLR model.

**SNPs that were exclusively or greater associated with men.

***SNPs that were exclusively or greater associated with women.

These 9 SNPs were analysed separately in women and men. Six SNPs were associated with T2D in men and only five in women (Table 4); four were common in both sexes (*SLC16A11*, *INS-IGF2*, *SLC30A8*, and *KCNQ1*). The SNP from *CDKN2B* was associated exclusively with women, whereas SNPs from *IGF2BP2* and *TCF7L2* were associated exclusively with men (Table 4). Interestingly, the r^2^ value was considerably higher in men than in women (r^2^ = 0.115 vs. r^2^ = 0.039), suggesting that genes account for a higher percent of the T2D variability in men than in women (11.5% vs. 3.9%).

To rule out if this difference is not an effect of the inequality in the number of samples between men (n = 952) and women (n = 1501), we built 100 different groups of 950 randomly selected women who were paired with men of the same age when they were diagnosed with T2D. For each sorted group we carried out MLR and then calculated the median r^2^. In the Supplementary Fig. S2, it can be observed that the mean r^2^ of the sorted sets of females (0.046) is increased only slightly compared to the r^2^ of the unsorted whole set of women (0.039), a value that remains still well below the r^2^ in males (0.115). This reinforces the observation that these genes have a greater effect on men than on women. On the other hand, the r^2^ increases from 0.065 to 0.076 in the total sample when exploring an equal number of individuals between men and women, which would seem to be a r^2^ value that is closer to reality.

**Figure 3:**
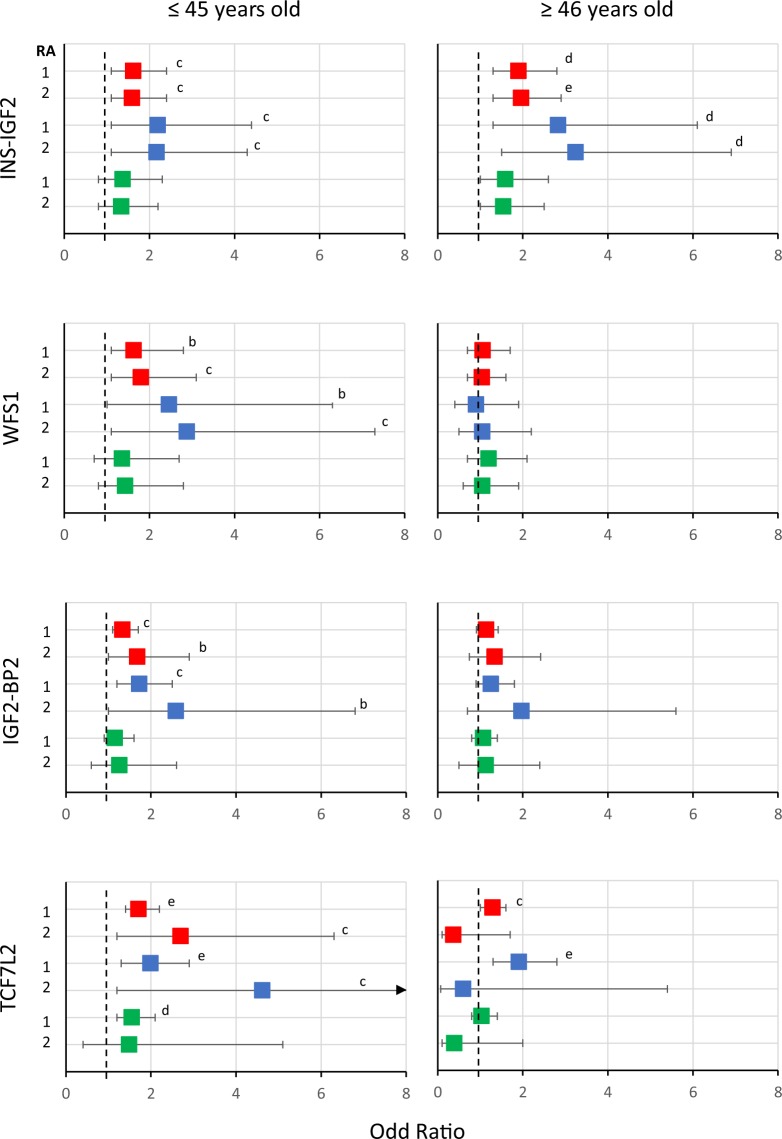
Odd ratios and 95%CI of genes associated exclusively or with a greater effect on men. ORs and 95% CI of genotypes with one or two risk alleles (RA) were calculated in multivariate logistic regression (MLR) models. The red squares indicate the values obtained for both sexes together, the blue squares the values for men and the green squares the values for women. The dotted line indicates the reference value (zero risk alleles), OR = 1. The arrow on the left panel of *TCF7L2* gene indicates that the higher limit of the confidence interval is outside the graph (12.5). The letters b, c, d and e indicate the values of p < 0.1, p < 0.05, p < 0.01, p < 0.001, respectively. The points that have no letters had a p > 0.1. The p-values were calculated in the MLR models with the Wald test. The data was taken from the Supplementary Table S8. The power (1-β err prob) >0.95 for all SNPs with a p < 0.1.

The role of the genes was also different between patients who were diagnosed with T2D at ≤45 years and ≥46 years of age. Eight SNPs were associated with a T2D diagnosis at ≤45 years old, whereas only six were associated with a T2D diagnosis at ≥46 years old. Five SNPs were common to both groups (from genes *SLC16A11*, *INS-IGF2*, *SLC30A8*, *KCNQ1*, and *TCF7L2*). Whereas *WFS1, IGF2BP2*, and *CDKN2B* were associated only with T2D diagnosed at ≤45 years of age, *ADCY5* was associated only with T2D diagnosis at ≥46 years of age (Supplemental Table S8). It is notable that the ORs of all but one (*INS-IGF2*) matching genes are much larger in the group diagnosed at a younger age (Supplemental Table S8). In fact, the r^2^ value was considerably higher in those who were diagnosed at ≤45 years of age than in those diagnosed at ≥46 years of age (r^2^ = 0.106 vs. r^2^ = 0.045, respectively), indicating that genes account for a higher percent of the T2D variability in the patients who were diagnosed at a younger age. When we stratified these groups by sex, it was clear that the influence of genes in men is much greater than in woman in both the ≤45 years (r^2^ = 0.182 vs. r^2^ = 0.077) and the ≥46 years diagnosis groups (r^2^ = 0.098 vs. r^2^ = 0.022). Similar figures were seen when the ORs were compared (Fig. 3). In the cross-sectional study, the r^2^ of genes was very similar to that in the main case-control study, at least in the global sample (6.8%). Similar to the main case-control study, the influence of genes is greater in men than in women (r^2^ = 0.034 vs. r^2^ = 0.308) (Supplementary Table S9). However, the number of cases of males (n = 30) and females (n = 70) was very small, well below the N recommended for MLR studies (see Material and Methods).

### Analysis of the total role of obesity, PH-T2D, and genes in T2D using an MLR model

**Figure 4:**
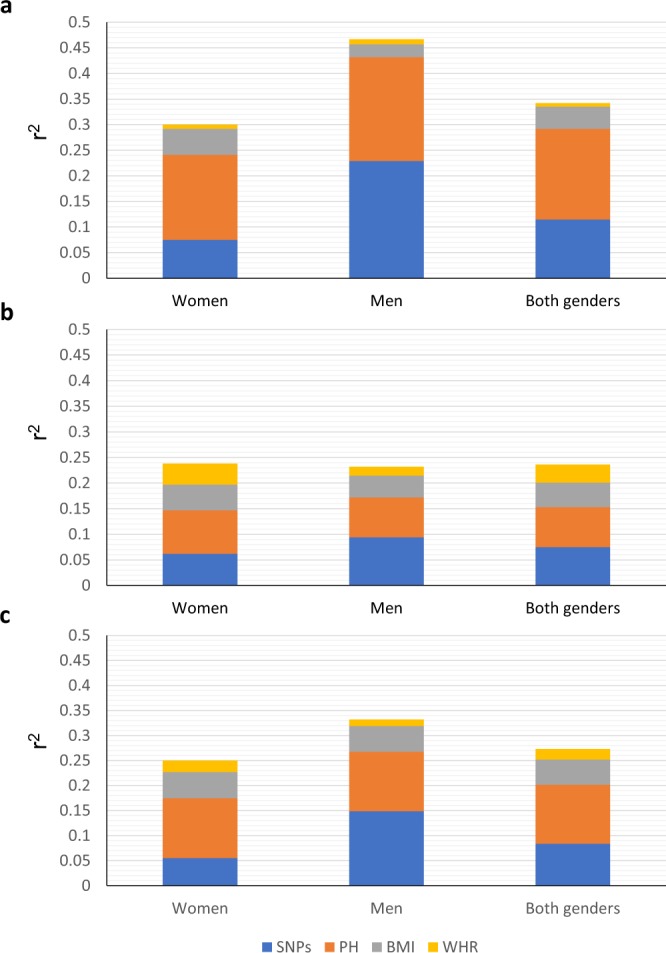
Influence (r^2^) of genes, PH of T2D and obesity in T2D. The effect of genes, parental history (PH) of type 2 diabetes (T2D) and obesity (BMI + WHR) on T2D, analysed by a multivariate logistic regression model (MLR) in the whole sample studied, and stratified by age at presentation of T2D and sex. In panel a the analysis was performed with cases diagnosed at ≤45 years of age (n = 326), in panel b, with cases diagnosed at ≥46 years of age (n = 432) and in the panel c, the analysis was performed with all cases included in this analysis (n = 763). For the 3 analyses, the same control group (n = 650), as a whole or stratified by sex, was included, depending on the comparison. This analysis included only the cases and controls that had complete data of the 4 factors analysed (n = 1413). The r^2^ values were obtained from Table 5. BMI = body mass index, WHR = ratio of waist/hip circumference. The power (1-β err prob) >0.95 for all multivariate logistic regression (MLR) models.

The total variability of T2D in Mexico accounted for by the factors included in this study (SNPs, PH, and obesity [adjBMI + WHR]) was 27.3% (Table 5). These three factors remained in the multivariate model, indicating that they play independent roles. The contribution of each factor was as follows: SNPs, 8.4%; PH, 11.8%; and obesity 7.1% (adjBMI, 5%; and WHR, 2.1%). However, the role of genes and PH is substantially higher when the disease is diagnosed at ≤45 years of age, and it decreases significantly when it is diagnosed at ≥46 years of age (Fig. 4). In contrast, the influence of obesity was slightly greater in T2D diagnosed after 46 years of age (Fig. 4). The total influence of these factors was higher when the disease was diagnosed at ≤45 years of age than when it was diagnosed at ≥46 years of age (r^2^ = 0.342 vs. r^2^ = 0.236, Table 5).

**Table 5:** Contribution of BMI, WHR, PH-T2D, and eight SNPs in T2D variability stratified by gender and age of disease presentation using MLR models*.

Variable	Block	Women				Men				Both genders			
		−2 log LH	r^2^	pb	pm	−2 log LH	r^2^	pb	pm	−2 log LH	r^2^	pb	pm
**Cases diagnosed at ≤45 years (n = 326)**													
SNPs	1	796	0.075	<0.0001	<0.0001	352	0.229	<0.0001	<0.0001	1159	0.115	<0.0001	<0.0001
PH	2	708	0.241	<0.0001	<0.0001	289	0.432	<0.0001	<0.0001	1013	0.292	<0.0001	<0.0001
BMI	3	678	0.292	<0.0001	<0.0001	281	0.457	0.003	<0.0001	974	0.335	<0.0001	<0.0001
WHR	4	674	0.300	0.037	<0.0001	277	0.467	0.06	<0.0001	967	0.342	0.009	<0.0001
**Cases diagnosed at ≥46 years (n = 432)**													
SNPs	1	927	0.062	0.002	0.002	469	0.094	<0.0001	<0.0001	1395	0.075	<0.0001	<0.0001
PH	2	878	0.147	<0.0001	<0.0001	445	0.172	<0.0001	<0.0001	1326	0.153	<0.0001	<0.0001
BMI	3	848	0.197	<0.0001	<0.0001	431	0.215	<0.0001	<0.0001	1281	0.201	<0.0001	<0.0001
WHR	4	822	0.238	<0.0001	<0.0001	426	0.232	0.019	<0.0001	1248	0.236	<0.0001	<0.0001
**All cases (n = 763)**													
SNPs	1	1251	0.055	<0.0001	<0.0001	602	0.149	<0.0001	<0.0001	1859	0.084	<0.0001	<0.0001
PH	2	1159	0.175	<0.0001	<0.0001	552	0.268	<0.0001	<0.0001	1718	0.202	<0.0001	<0.0001
BMI	3	1116	0.227	<0.0001	<0.0001	528	0.319	<0.0001	<0.0001	1654	0.252	<0.0001	<0.0001
WHR	4	1097	0.25	<0.0001	<0.0001	522	0.332	0.014	<0.0001	1627	0.273	<0.0001	<0.0001

r^2^ (Nagelkerke) and p-values (omnibus test) were calculated with the multivariate logistic regression models.

LH = likelihood, PH = parental history of T2D; BMI = adjusted body mass index; WHR = waist/hip ratio; pb = p-value of the block; pm = p-value of the model.

*Only the cases (n = 763) and controls (n = 650) that had the data complete for the 4 factors (n = 1413) were included in the analysis. For the group of all cases or groups of cases diagnosed at ≤45 or at ≥46 years, the same control group, as a whole or stratified by sex, was included in the analysis, depending on the comparison. The power (1-β err prob) >0.95 for all multivariate logistic regression (MLR) models.

As we observed previously in the ULR models, when the two genders were analysed separately, the contribution of each factor was different. The contribution of SNPs was much greater in men than in women (14.9% vs. 5.5%), whereas the role of PH and BMI was similar in women and men (Fig. 4c). Therefore, the total contribution of these factors was higher in men than in women (33.2% vs. 25%, respectively), particularly when the disease was diagnosed before the age of 46 (46.7% vs. 30%). This difference was mainly due to the greater difference in the contribution of genes in younger patients (Fig. 4). No interactions were found among the factors. All nine but one genes (*ADCY5*) associated with T2D in the MLR model that was run with only genes also remained associated with the disease in the MLR model that used the rest of the factors. Smoking and sedentariness did not contribute to T2D in either the ULR or MLR models.

## Discussion

In this study, we demonstrated that SNPs, PH, and obesity (adjBMI + WHR) accounted for only 27.3% of the variability of T2D in Mexico, and the contribution of each factor was 8.4%, 11.8%, and 7.1% (BMI 5%, and WHR 2.1%), respectively. However, the contribution of these factors was greater in men than in women (33.2% vs. 25%, respectively), particularly when the disease was diagnosed before the age of 46 (46.7% vs. 30%, respectively). Genes played a substantially more important role in men than in women, while obesity and PH played a similar role in both genders. The genes *SLC16A11*, *INS-IGF2*, *KCNQ1*, and *SLC30A8* were associated with both genders, whereas *CDKN2B* were associated exclusively with women, and *WFS1*, *IGF2BP2*, and *TCF7L2* exclusively with men.

The main limitation of this work is that the BMI of patients with T2D at the time of recruitment did not appear to be the same as the BMI at the time of diagnosis. Therefore, the measurement of the contribution of BMI on the variability of T2D (r^2^ = 0.028) appears to differ from what is seen in a real-life setting^30^. This result corresponds with other studies in Mexico in which no association^21,31^, or a poor association^21,32^, was found between BMI and T2D. Although the adjustment made to the BMI using the 100th-percentiles of the BMI of patients with ≤2 years with the disease substantially increased the r^2^ value (0.126), which is close to the value measured in the cross-sectional study (r^2^ = 0.138) when all cases were included in the analysis, this value may still be very different from the actual contribution of obesity to the disease^30^. In fact, when only the newly diagnosed cases were included in the analysis in the cross-sectional study, the value of r^2^ (0.149) was further separated from the r^2^ of the adjusted BMI. However, even this value could be far from the actual influence of the BMI on T2D, because the disease most likely begins several years before diagnosis when obesity possibly plays a greater role in the development of insulin resistance. A defective insulin metabolism decreases the synthesis of lipids and proteins and facilitates energetic catabolism, which may explain why patients lose weight over time. A similar pattern of weight loss has also been observed in other studies^33,34^.

Another limitation of the study is that the number of cases (n = 104) and controls (n = 204) in the cross-sectional study is very small. However, the cross-sectional study, even with this small sample, allowed us to gain an understanding of the age at which patients with T2D are diagnosed, the effect of the BMI and to a lesser extent, the influence of parental history of T2D and genes on the variability of T2D in another sample population of Mexico. Although the r^2^ value calculated for the BMI and genes was very similar to that calculated in the main case-control study, the r^2^ value of PH was much smaller in the cross-sectional study. The age at diagnosis of the patients in the case-control studies was three to four years younger than of those included in the cross-sectional study or that was reported in other studies based on population sampling^35^. This difference seems to be associated with the fact that the hospital cases, who have social security, are diagnosed earlier than the general population cases^36^. The inclusion of a group of patients with an earlier presentation of the disease may have contributed to a stronger association between T2D and PH or SNPs than between BMI and the disease.

Our findings clearly showed that the role played by the studied factors (obesity, PH, and genes) in T2D variability is not the same across all age groups in Mexico. While the influence of the parental history of diabetes and genes decreased as the age of T2D presentation increased, the influence of obesity remained more or less constant at all ages of the disease presentation.

It is not surprising that the BMI contributes independently from PH and genes to T2D in Mexico, specifically because it is an environmental factor that does not usually depend on family or genetic factors, but rather on poor eating and lifestyle habits. Of particular interest is that PH and genes contributed independently to T2D in Mexico. Similar results have been found in a study on European populations^37^. This suggests that there are many other genes involved that were not included in this study and that there are other family factors transmitted to the offspring, such as inherited epigenetic factors, that are not directly related to SNPs. Alternatively, T2D is a complex disease that may depend on many factors, and the disease does not always manifest when the RAs or PH factors are present, as would be the case for the controls that have RAs or have a positive family history.

The considerable difference in the association of genes between men and women was unexpected. Furthermore, genes had a larger influence in early (≤45 years old) than in late (≥46 years old) presentation of T2D in men, but not in women. Since none of the explored genes in this study are located on the sex chromosomes, the differences observed between men and women are unlikely to be a result of sexual chromosome segregation. Instead, it would suggest that the functions of the studied genes vary due to the different hormonal influences between men and women. Therefore, we would expect to find some relationship between testosterone and the genes associated exclusively (*WFS1*, *IGF2BP2*, and *TCF7 L2*) or with a greater effect (*INS-IGF2*) in men, as well as between female hormones and the gene associated exclusively with women (*CDKN2B*). Many other genes in addition to those described above have been found to be associated differently between women and men in a European cohort^38,39^, which supports our finding that some genes may be associated differently between genders. The biggest difference in the effect of genes between men and women is observed in the early presentation of the disease, before menopause, suggesting that a hormonal effect may protect or delay the impact of genes on T2D development in women. In support of this hypothesis, it has been demonstrated that hormone therapy reduces the incidence of diabetes by 35% in postmenopausal women with coronary heart disease compared with a placebo group^40^ and that early menopause is associated with a greater risk of T2D^41^.

Interestingly, there is solid evidence that the *TCF7L2* gene behaves differently in women and men. In a large study of cases and controls in a European population, it was found that the SNPs of this gene, which were individually explored, were associated with T2D only in men, but when they were explored altogether, the gene was also associated with T2D in women, although in this case the association was also greater in men^42^. In fact, the gene *TCF7L2* makes a strong contribution to the significance of the metabolic pathways associated with T2D in men, but not in women^42^. *TCF7L2* is a master regulator of insulin production and processing, and the risk T-allele of rs7903146 was associated with increased *TCF7L2* expression and decreased insulin content and secretion^43^. The association of *IGF2BP2* rs4402960 with T2D in the Mexican population was replicated in this study. However, this is the first study in which this SNP was associated with T2D in men, but not in women. Interestingly, in a previous study in a European population, this SNP was found to be associated with diabetic nephropathy in men but not in women with type 1 diabetes^44^. It is clear from our data that the effect of *TCF7L2* and *IGF2BP2* on men is much greater when the disease is diagnosed at ≤45 years of age than when it is diagnosed at an older age, especially for the homozygous risk allele (OR = 4.62 and 2.59, respectively; Fig. 3). The *WFS1* gene (rs4458523) was also found to be associated with T2D exclusively in men, but only when they were diagnosed at ≤45 years of age. This gene has not been reported to be associated with T2D in men only. However, there is a mouse study that found that the deletion of the gene *WFS1* increases the risk of T2D only in male mice^45^. On the other hand, the association of this gene with the development of early onset T2D in that paper agrees with previous reports in which mutations in this gene were associated with the development of juvenile-onset diabetes mellitus. Remarkably, in those cases, the onset of diabetes tended to occur earlier in boys than in girls^46^. In general, worldwide T2D is diagnosed at an earlier age in males than in females^39^. Therefore, the *TCF7L2*, *IGF2BP2* and *WFS1* genes, which are all involved in the production, maturation or secretion of insulin, may contribute to this difference between men and women.

Polymorphism of the SNP rs149483638, located in the genes INS-IGF2 and IGF2, which are very close to the insulin gene (INS), does not exist or is rare in populations other than Hispanic (type2diabetesgenetics.org.2018 Nov 8; http://www.type2diabetesgenetics.org). Therefore, this is the first time that the effect of the risk allele has been reported to be much more important in men than in women; in fact, the association in women disappears when the sample is stratified by age of disease presentation. The effect of the gene on males is greater when the disease is diagnosed at ≥46 years of age. Interestingly, these are imprinted genes, expressed only from the paternal allele^47^. Although in our study the inheritance of the mother was greater than that of the father, it is very likely that for the locus INS-IGF2, the risk allele in men is inherited from the paternal side.

Similar to other studies^48–50^, maternal inheritance was more important than paternal inheritance in the present study. We could not rule out that the mother’s greatest influence is due to biological factors, mainly because in both case-control studies, the maternal and paternal influence (r^2^) fall linearly and in parallel as the age at which patients were diagnosed increases (Fig. 2). These trends suggest a biological rather than a confounding effect. Although we cannot rule out epigenetic factors transmitted by the mother^51^, the data suggest that genetic factors linked to the X chromosome or mitochondrial DNA may be associated with the greater genetic influence of the mother in the T2D in Mexico. Since during the 300 years of colonization in Mexico sexual interchanges took place mainly between Spanish men and Amerindian women^52^, at least two thirds of X chromosome-linked genes compared with only 50% of autosome-linked genes, and all mitochondrial genes, originated from Amerindian populations. However, it is clear to us that it is necessary to validate some of the results found in this paper in larger samples in Mexico and in other world populations.

## Materials and Methods

### Sample selection and study design

The individuals included in the main case-control study form part of the Diabetes in Mexico Study (DMS), the design of which has been described previously as part of the SIGMA Type 2 Diabetes Consortium^23,24^. In brief, the participants were recruited from two tertiary level hospitals in Mexico City: Centro Médico Nacional siglo XX1 from the Mexican Social Security (IMSS) and Centro Médico Nacional 20 de Noviembre from the Social Security for State Workers (ISSSTE). T2D was diagnosed based on the criteria of the American Diabetes Association (ADA). A total of 1250 cases (unrelated individuals, over 20 years of age, with a previous diagnosis of T2D or fasting glucose levels above 125 mg/dL) and 1250 controls (healthy subjects over 50 years of age and with fasting glucose levels below 100 mg/dL) were selected from the DMS database, which consists of 1500 controls and 1500 cases of the Mestizo population. These cases and controls (1:1) were recruited from November 2009 to August 2013. For each recruited case, one control of the same sex and from the same hospital was recruited. Clinical information was collected during the initial interview, including weight, height, waist and hip circumference, and PH of T2D. For fasting glucose measurements and DNA extraction, 10 mL of intravenous blood was collected. All participants were confirmed to be of Mexican mestizo ancestry according to self-reporting and genotypic analysis^24^.

For the replication of the analysis of clinical variables (BMI, waist and hip circumferences, waist/hip ratio (WHR) and PH of T2D) a clinical replica case-control study was designed, including a total of 2489 prevalent cases of T2D and 415 controls. They were collected from the Family Medicine Units numbers 31, 10, 15 and 23 of the IMSS of Mexico City between January 2014 and January 2015. The cases, who were previously diagnosed with T2D according to the international criteria (ADA) and who agreed to enter the study, were continuously recruited during the periodical medical revision. The controls recruited were individuals who attended the same clinics for reasons other than T2D, agreed to enter the study, had a fasting glucose level of less than 100 mg/dL and were ≥50 years old. A clinical history and measurements of the anthropometric and biochemical parameters were carried out in all the patients and controls.

For replication of molecular and clinical data, a population-based cross-sectional study was conducted. A total of 2005 individuals were recruited between July and December 2017 from the Hospital General de Puebla “Ignacio Romero Vargas” located in the suburb area of the city of Puebla, Mexico. The recruited individuals were healthy people who were invited to participate in the study by distributing flyers house-to-house in the neighbourhood in which the hospital is located. Once in the clinic, the percentage of acceptance to participate in the study was 85%, which is a very similar percentage to those reported in other studies in Mexico. The participants were surveyed to explore T2D risk factors and weighed and measured for height and waist circumference. A blood sample was also taken to measure glycated haemoglobin (A1c) and to explore the polymorphism of 16 SNPs associated with T2D. Individuals who had an A1c ≥ 6.5 were considered diabetic, and those who had an A1c < 5.7 were considered non-diabetic.

The association of BMI, waist (W) and hip (H) circumferences, WH ratio (WHR), PH of T2D, and the 16 selected genes associated with T2D were investigated using ULR and MLR models. For the analysis, the diagnosis of T2D or the control was considered the dependent variable, and the values of the single nucleotide polymorphisms (SNPs) genotypes, PH, and BMI were considered as the explanatory variables. Genotypes and PH were classified as 0, 1, or 2, according to the number of risk alleles (RA) and the number of parents with T2D. BMI was considered as either a continuous or an ordinal variable, depending on the type of analysis. The main case-control study was approved by the Ethics and Research Committees of the Instituto Nacional de Medicina Genómica and the Federal Commission for the Protection against Health Risks (COFEPRIS), with the number CAS/OR/CMN/113300410D0027-0577/2012. The clinical replica study was approved with the number IMSS R-2014-785-005 by the Ethics and Research Committees of the Comisión Nacional de Investigación Científica of the IMSS, and the cross-sectional study was approved with the number 68/ENS/INV/REV/2017 by the Ethics and Research Committees of the Hospital General de Puebla “Ignacio Romero Vargas”. All three protocols were performed in accordance with the ethical principles described in the 1964 Declaration of Helsinki. Informed written consent was obtained from all participants prior to their inclusion in the study.

### SNP selection and genotyping

We selected 16 SNPs using the largest meta-analyses of GWAS available in diabetic patients, prioritizing those with the highest predictive power (effect size × allele frequency) in the Latin American mestizo population and that were localized in genes from various cellular processes involved in T2D development: solute carrier family 16 member 11 (*SLC16A11*), INS-IGF2 readthrough (*INS-IGF2*), HNF1 homeobox A (*HNF1A*), wolframin ER transmembrane glycoprotein (*WFS1*), solute carrier family 30 member 8 (*SLC30A8*), peroxisome proliferator activated receptor gamma (*PPARG*), insulin-like growth factor 2 mRNA binding protein 2 (*IGF2BP2*), CDK5 regulatory subunit-associated protein 1-like 1 (*CDKAL1*), adenylate cyclase 5 (*ADCY5*), JAZF zinc finger 1 (*JAZF1*), haematopoietically expressed homeobox (*HHEX*), potassium voltage-gated channel subfamily J member 11 (*KCNJ11*), potassium voltage-gated channel subfamily Q member 1 (*KCNQ1*), transcription factor 7-like 2 (*TCF7 L2*), alpha-ketoglutarate dependent dioxygenase (*FTO*), and cyclin-dependent kinase inhibitor 2B (*CDKN2B*) (Supplementary Table S6). All DNA samples of the main case-control study and cross-sectional study were genotyped for the SNPs listed in Supplementary Table S6. Assays were designed using the Applied Biosystems (Foster City, CA, USA) TaqMan SNP assay design technology. Genotyping was performed by the allelic discrimination assay-by-design TaqMan® method on OpenArray® plates. The plates were analysed on the QuantStudio™ 12 K Flex Real-Time PCR System (ThermoFisher Scientific, Waltham, MA, USA). The genotypes were analysed by Genotyper™ Software v1.3 (ThermoFisher Scientific, Waltham, MA, USA).

### Statistical analyses

The patient age, age at T2D diagnosis, BMI, waist and hip circumferences and WHR results were expressed as the means ± standard deviation (SD) or as the median and interquartile range (IQR, 25–75%). To assess the statistical significance of differences between the groups, the *t* test was performed for mean values, and the Mann-Whitney U-test was used for median values. We investigated whether the frequency of genotypes was distributed according to the Hardy-Weinberg law based on the allelic frequency and the formula: (*a* + *b*)^2^ = *a*^2^ + 2ab + *b*^2^, where *a* and *b* are the allelic frequencies in the control group. The statistical significance of the differences in the distribution of genotypes between the observed and expected results was calculated using the chi-squared test.

The trend of BMI was analysed according to the number of years with T2D, and the significance of the correlation was calculated using the Pearson correlation test. In certain calculations, the BMI was adjusted (adjBMI) for patients with ≥3 years with T2D using data from patients with ≤2 years with the disease as follows: the 100th-percentile values of the BMI were calculated for both groups, and two equations were derived between the BMI values and the 100th-percentiles rank data. Next, the exact 100th-percentile was calculated for each BMI value of the group to be adjusted using the equation derived from that group (*y* = 1.7009ln(*x*) −5.1786, [r = 0.98]), where *y* = 100th-percentile and *x* = the original BMI value. The adjBMI was then calculated using the equation derived from the cases with ≤2 years with the disease (*y* = 22.99*e*^0.5675*x*^, [r = 0.97]), where *y* = adjBMI, and *x* = the calculated 100th-percentile value of the dataset to be adjusted. A similar procedure was used to adjust the waist and hip circumferences. When BMI was analysed as a categorical variable, it was grouped into <25, 25–30, and >30 kg/m^2^, based on the World Health Organization BMI categories.

The analysis of all variables (BMI, PH, and SNPs) was performed in the entire sample and stratified by sex and the median age at T2D diagnosis. The risk conferred by each factor (explanatory variables) was calculated by comparing cases and controls using ULR models. The reference indicators of the explanatory variables BMI, PH of T2D, and SNPs, were BMI <25, no parents with T2D, and genotypes with no risk alleles (RA), respectively. The BMI, waist, hip and WHR was also entered into the models as a continuous variable. The association was expressed as the OR and 95% confidence interval (CI), and the contribution to the variability of T2D was expressed as Nagelkerke’s r^2^. Confounders were identified using a theoretical strategy based on a backstep, stepwise MLR model and the change-in-estimate criterion. Variables with p < 0.20 in the univariate analysis were considered for entry in the multivariate model. Confounders were defined as those variables for which the percentage difference between the values of the regression β between the adjusted and non-adjusted variables in the stepwise multivariate model was larger than 10% (p > 0.1). Therefore, the total variability, the contribution of each factor, and interaction between the factors on T2D was calculated using this MLR model. However, the MLR model was performed in strata only if the sample size included at least 15 cases for each variable introduced into the model^53^ and always (as in the ULR models) using a minimum of 50^54^ or 100 cases^55^. The factors and interactions were included successively in the model in different blocks. The contribution of each factor to the model was assessed by the increase of r^2^ and the decrease in the −2 log likelihood ratio (LH) value from one to the next block; the Omnibus test was used to determine whether the differences between the successive blocks were statistically significant. A post hoc power analysis was performed for each logistic regression model using the software G * Power See 3.1.9.2, considering the sample size, the OR, the probability of the event in the control group, and an α = 0.05^56^. In addition, for MLR models, the value of the total r^2^ obtained at the end of the model was introduced for power calculation. All statistical tests were two-sided, and differences were considered significant when p < 0.05. The statistical analyses were conducted using SPSS version 20 software (SPSS Inc., Chicago, IL, USA).

## Supplementary information

Supplementary Tables and Figures

## Data Availability

All data generated or analysed during this study are included in this article.
