# Supplementary material for: Influence of obesity, parental history of diabetes, and genes in type 2 diabetes: A case-control study
Source: Sci Rep. 2019 Feb 26;9:2748. doi: 10.1038/s41598-019-39145-x (PMC6391418; doi:10.1038/s41598-019-39145-x)
Supplement: Supplementary file 1 — Supplementary Tables and Figures [file 41598_2019_39145_MOESM1_ESM.pdf]

Influence of obesity, parental history of diabetes, and genes in type 2 diabetes: A case-control study

Jaime Berumen[1,2*], Lorena Orozco[3], Miguel Betancourt[4], Héctor Gallardo[4], Mirella Zulueta[5], Leire Mendizabal[5], Laureano Simon[5], Rosa Benuto[6], Elisa Ramírez-Campos[6], Melissa Marin[6], Eligia Juárez[1], Humberto García-Ortiz[3], Angélica Martínez-Hernández[3], Carlos Venegas-Vega[7], Jesús Peralta-Romero[8], Miguel Cruz[8], Roberto Tapia-Conyer[4]

Supplementary Table S1. Selected clinical characteristic of clinical replica case-control study

Variable	Women (1867)		Men (1037)		Both genders (2904)	
	**Control** (n=280)	**Cases** (n=1587)	**Control** (n=135)	**Cases** (n=902)	**Control** (n=415)	**Cases** (n=2489)
Continuous variables: means ± SD						
Age (years)	59.6 ± 7.3	56.6 ± 9[e]	61.1 ± 8.1	57.4 ± 10.6[e]	60.1 ± 7.6	56.9 ± 9.6[e]
BMI (kg/m^2)	28.1 ± 4.6	30.1 ± 5.1[e]	28.1 ± 4.2	28.3 ± 4.5[a]	28.1 ± 4.5	29.4 ± 5[e]
Waist (cm)	90.2 ± 10.4	96.1 ± 12.9[e]	96.6 ± 13.7	96.2 ± 13[a]	92.3 ± 12	96.1 ± 13[e]
Hip (cm)	103.3 ± 11	104.5 ± 27.8[a]	101.1 ± 7.7	105.9 ± 35.9[a]	102.6 ± 10.1	105 ± 31[a]
WHR (Waist/Hip)	0.87 ± 0.07	0.93 ± 0.09[e]	0.95 ± 0.1	0.92 ± 0.09[e]	0.9 ± 0.09	0.92 ± 0.09[e]
Age at diabetes diagnosis (years)		45.6 ± 7.2		45 ± 7.7		45.4 ± 7.4
Years with the disease		11 ± 8.2		12.4 ± 9.1		11.5 ± 8.6
Parental diabetes history: % (n)*						
None	63.6 (178)	35.7 (560)[e]	63 (85)	38.7 (345)[e]	63.4 (263)	36.8 (905)[e]
Father	15.7 (44)	32.3 (506)	20 (27)	32.2 (287)	17.1 (71)	32.2 (793)
Mother	24.6 (69)	48.9 (767)	21.5 (29)	43.6 (389)	23.6 (98)	47 (1156)
Both parents	3.9 (11)	16.9 (265)	4.4 (6)	14.5 (129)	4.1 (17)	16 (394)
Total	100 (280)	100 (1568)	100 (135)	100 (892)	100 (415)	100 (2460)

BMI, waist and hip circumferences shown are those measured at enrollment.

For the differences of means between the groups the p-values were assessed using the *t* test

For the differences in the frequency distribution between the groups the p-values were assessed with the chi-square test.

The superscripts indicate the p-values as follows: [a]p>0.05, [b]p<0.05, [c]p<0.01, [d]p<0.001 and [e]p<0.0001

*The sum of the percent of None, Father and Mother less the percent of both parents give 100%.

Supplementary Table S2. Selected clinical characteristic of population-based cross-sectional study (n=1914)

Variable	Cross-sectional study [a]				
	**Controls ≥ 50 years** (n=204)	**Controls ≤ 49 years** (n=1071)	**Pre diabetics** (n=535)	**Cases** (n=104)	**p value**[b]
Continuous variables: means ± SD					
Age (years)	58.7 ± 7.9	34.7 ± 8.6	48.5 ± 12.3	49.2 ± 11.4	<0.0001
BMI (kg/m^2)	27.7 ± 4.2	26.7 ± 4.5	29.2 ± 4.6	31 ± 5.5	<0.0001
Waist (cm)	91.7 ± 10.2	88.7 ± 13.9	93.2 ± 17.4	99.2 ± 14.6	<0.0001
Age at diabetes diagnosis (years)				49.5 ± 11.3[c]	
Years with the disease				0	
Parental diabetes history: % (n)*					
None	64.2 (131)	60.6 (649)	56.1 (300)	52.9 (55)	0.06
Siblings only	5.4 (11)	4.0 (43)	5.6 (30)	3.8 (4)	
Parents only	24.5 (50)	29.2 (313)	31.6 (169)	34.6 (36)	
Both parents and siblings	5.9 (12)	6.2 (66)	6.7 (36)	8.7 (9)	
Total	100 (204)	100 (1071)	100 (535)	100 (104)	
Smoking: % (n)					
No	89.2 (181)	83.6 (895)	86 (460)	82.7 (86)	0.08
Yes	10.8 (22)	16.4 (176)	14 (75)	17.3 (18)	
Total	100 (203)	100 (1071)	100 (535)	100 (104)	
Sedentary lifestyle: % (n)					
No	72.8 (147)	67.2 (720)	67.7 (362)	72.1 (75)	>0.05
Yes	27.2 (55)	32.8 (351)	32.3 (173)	27.9 (29)	
Total	100 (202)	100 (1071)	100 (535)	100 (104)	

BMI and waist circumferences shown are those measured at enrollment.
For the differences of means between the groups the p-values were assessed using the *t* test
For the differences in the frequency distribution between the groups the p-values were assessed with the chi-square test
a. The mean age of the total control group was 38.6 ± 12.2 (n=1275)
b. The p-value was calculated for comparisons between cases and the controls ≥ 50 years
c. The mean was calculated with individuals which were newly diagnosed with T2D during the study (n=92).

Supplementary Table S3. Association of raw and adjusted BMI with T2D stratified by gender using ULR models in the main case-control study*

Varaiable	Controls (n)	Raw BMI					Adjusted BMI				
		Cases (n)	r^2	p-value (Omnibus)	OR (95CI)	p-value (wald)	Cases (n)	r^2	p-value (Omnibus)	OR (95CI)	p-value (wald)
**Women**											
BMI[a]	755	737	0.019	<0.001	1.047 (1.027 - 1.068)	<0.001	737	0.103	<0.001	1.12 (1.09 - 1.14)	<0.001
≤ 24.9	199	144			1		83			1	
25-29.9	314	284	0.016	<0.001	1.25 (0.956 - 1.634)	>0.05	240	0.093	<0.001	1.81 (1.34 - 2.46)	<0.001
≥ 30	242	309			1.765 (1.344 - 2.317)	<0.001	414			4.19 (3.09 - 5.66)	<0.001
**Man**											
BMI[a]	469	472	0.049	<0.001	1.091 (1.059 - 1.124)	<0.001	472	0.181	<0.001	1.2 (1.16 - 1.24)	<0.001
≤ 24.9	145	117			1		57			1	
25-29.9	226	184	0.038	<0.001	1.009 (0.739 - 1.378)	>0.05	178	0.141	<0.001	2 (1.39 - 2.88)	<0.001
≥ 30	98	171			2.162 (1.527 - 3.062)	<0.001	237			6.15 (4.18 - 9.05)	<0.001
**Both sex**											
BMI[a]	1224	1209	0.028	<0.001	1.06 (1.043 - 1.077)	<0.001	1209	0.126	<0.001	1.14 (1.12 - 1.16)	<0.001
≤ 24.9	344	261			1		140			1	
25-29.9	540	468	0.022	<0.001	1.142 (0.932 - 1.399)	>0.05	418	0.109	<0.001	1.89 (1.5 - 2.39)	<0.001
≥ 30	340	480			1.861 (1.504 - 2.301)	<0.001	651			4.77 (3.77 - 6.05)	<0.001

a. BMI introduced in the model as a continue variable
r^2 (Nagelkerke)
*The power (1-β err prob) >0.95 for all univariate logistic regression (ULR) models.

Supplementary Table S4. Association of raw BMI, waist and Hip circumferences and Waist/Hip ratio with T2D stratified by the number of years with T2D using ULR models*

Variable	Controls	Cases								
		≤ 2 years with the disease			> 2 years with the disease			All		
	n	n	r^2	p[a]	n	r^2	p[a]	n	r^2	p[a]
**BMI**										
Women	755	189	0.066	<0.001	544	0.007	0.008	737	0.019	<0.001
Men	469	109	0.174	<0.001	361	0.020	<0.001	472	0.049	<0.001
Both sex	1224	298	0.096	<0.001	905	0.010	<0.001	1209	0.028	<0.001
**Waist**										
Women	567	144	0.057	<0.001	452	0.034	<0.001	600	0.043	<0.001
Men	315	59	0.112	<0.001	226	0.052	<0.001	287	0.067	<0.001
Both sex	882	203	0.072	<0.001	678	0.040	<0.001	887	0.05	<0.001
**Hip**										
Women	541	143	0.034	<0.001	451	0.015	0.001	597	0.021	<0.001
Men	303	58	0.121	<0.001	223	0.012	0.029	283	0.029	<0.001
Both sex	844	201	0.057	<0.001	674	0.015	<0.001	880	0.024	<0.001
**Waist/Hip ratio**										
Women	541	143	0.016	0.007	450	0.014	0.001	596	0.016	<0.001
Men	303	58	0.036	0.005	223	0.080	<0.001	283	0.076	<0.001
Both sex	844	201	0.015	0.002	673	0.024	<0.001	879	0.023	<0.001

r^2 = Nagelkerke method

a. Omnibus test

*The power (1-β err prob) >0.95 for all univariate logistic regression (ULR) models.

Supplementary Table S5. Association of adjusted waist and hip circumferences and non-adjusted waist/hip ratio with T2D stratified by sex using ULR models*

Variable	Controls (n)	Cases (n)	r^2	p^a	OR (95CI)	p^b
**adjWaist**						
Women	567	600	0.083	<0.001	1.05 (1.04 - 1.06)	<0.001
Men	315	287	0.117	<0.001	1.06 (1.04 - 1.08)	<0.001
Both sex	882	887	0.093	<0.001	1.05 (1.04 - 1.06)	<0.001
**adjHip**						
Women	541	597	0.059	<0.001	1.04 (1.03 - 1.05)	<0.001
Men	303	283	0.093	<0.001	1.07 (1.04 - 1.09)	<0.001
Both sex	844	880	0.069	<0.001	1.04 (1.03 - 1.05)	<0.001
**Waist/Hip ratio**						
Women	541	596	0.016	<0.001	1.03 (1.02 - 1.05)	<0.001
Men	303	283	0.076	<0.001	1.09 (1.06 - 1.12)	<0.001
Both sex	844	879	0.023	<0.001	1.04 (1.03 - 1.06)	<0.001

r^2 = Nagelkerke method

a. Omnibus test

b. Wald test

*The power (1-β err prob) >0.95 for all univariate logistic regression (ULR) models.

Supplementary Table S6. SNPs associated with T2D explored in this study, including the risk allele frequency (RAF) and OR reported in Mexican populations (n=4906)

Gene (SNP)	Chr (Position[a])	Alleles[b]		Mexican[c]			Present study		
		Risk	Alt	RAF	OR	p-value	RAF	OR (CI)*	p-value
SLC16A11 (rs75493593)	17 (7041768)	T*	G	0.30	1.29	$5.5x10^{-12}$	0.396	1.18 (1.05 - 1.32)	0.004
INS-IGF2 (rs149483638)	11 (2140300)	C	T*	0.84	1.28	1.17E-07	0.701	1.26 (1.11 -1.43)	<0.001
HNF1A (rs483353044)	12 (120999288)	A*	G	0.02	5.48	$4.4x10^{-7}$	0.004	2.15 (0.97 - 4.75)	0.06
WFS1 (rs4458523)	4 (6288259)	G*	T	0.26	1.12	1.26E-03	0.777	1.02 (0.89 - 1.17)	>0.1
SLC30A8 (rs3802177)	8 (117172786)	A*	G	0.256	0.890	1.15E-04	0.307	1.21 (1.08 - 1.37)	0.002
PPARG (rs1801282)	3 (12351626)	C	G*	0.91	1.10	$4.0x10^{-2}$	0.828	1.17 (1 - 1.36)	0.047
IGF2BP2 (rs4402960)	3 (185793899)	T*	G	0.24	1.11	3.30E-03	0.160	1.27 (1.09 - 1.47)	0.002
CDKAL1 (rs7756992)	6 (20679478)	G*	A	0.32	1.06	7.35E-02	0.329	0.95 (0.84 - 1.07)	>0.1
ADCY5 (rs11717195)	3 (123363551)	T	C*	0.69	1.12	7.09E-04	0.587	1.17 (1.05 - 1.32)	0.006
JAZF1 (rs849135)	7 (28156794)	G	A*	0.66	1.15	3.90E-05	0.714	1.05 (0.92 - 1.19)	>0.1
HHEX (rs1111875)	10 (92703125)	T*	C	0.374	0.946	8.76E-02	0.388	0.93 (0.83 - 1.04)	>0.1
KCNJ11 (rs5219)	11 (17388025)	T	C*	0.6	1.08	2.00E-02	0.393	1.08 (0.96 - 1.21)	>0.1
KCNQ1 (rs2237897)	11 (2837316)	C	T*	0.74	1.31	2.40E-13	0.603	1.51 (1.34 - 1.7)	<0.001
TCF7L2 (rs7903146)	10 (112998590)	T*	C	0.21	1.37	8.35E-16	0.113	1.43 (1.21 - 1.68)	<0.001
FTO (rs9936385)	16 (53785257)	C*	T	0.23	1.15	1.71E-04	0.130	1.24 (1.06 - 1.45)	0.009
CDKN2B (rs10811661)	9 (22134095)	T	C*	0.89	1.08	1.42E-01	0.906	1.19 (0.98 - 1.45)	0.083

Alt, alternate allele; NC, not calculated; OR, odds ratio; RA, risk allele; RAF, risk allele frequency; T2D, diabetes mellitus type 2

a. The position of SNPs is based on the human genome assembly GRCh38.

b. The variant allele is labeled with an asterisk and the ancestral allele or the reference in the human genome sequence is unlabeled.

c. Reported data for European (70K for T2D GWAS, 19K exome) and Mexican (GWAS SIGMA) populations were obtained from the AMP-T2D Program; T2D-GENES Consortium, SIGMA T2D Consortium; type2diabetesgenetics.org. 2018 Jul 30; http://www.type2diabetesgenetics.org.

*The power (1-β err prob) >0.95 for all univariate logistic regression (ULR) models with a p<0.1.

Supplementary Table S7. Genotypic frequencies and ORs of 16 SNPs associated with T2D in a sample of cases and controls in Mexico City (n=2453)[a]

Gene (SNP)	Number of Risk Alleles	Genotypic frequency % (n)			Multivariate logistic regression model	
		Controls (n=1234)	Cases (n=1219)	p-value	OR (95% CI)	p-value
SLC16A11* (rs75493593)	0	39.5 (487)	32.5 (396)	0.001	1	8.96E-05
	1	41.8 (516)	47.7 (581)		1.45 (1.21 - 1.74)	7.37E-05
	2	18.7 (231)	19.9 (242)		1.48 (1.17 - 1.86)	9.86E-04
INS-IGF2* (rs149483638)	0	10.9 (135)	6.3 (77)	<0.001	1	1.17E-03
	1	37.8 (467)	37.9 (462)		1.76 (1.29 - 2.42)	4.19E-04
	2	51.2 (632)	55.8 (680)		1.75 (1.28 - 2.38)	4.13E-04
HNF1A* (rs483353044)	0	99.3 (1225)	98.5 (1201)	>0.05	1	
	1	0.7 (9)	1.5 (18)		1.89 (0.8 - 4.3)	9.80E-02
WFS1 (rs4458523)	0	5.4 (67)	4.6 (56)	>0.05	1	>0.1
	1	33.8 (417)	34.8 (424)		1.26 (0.85 - 1.86)	>0.1
	2	60.8 (750)	60.6 (739)		1.29 (0.88 - 1.89)	>0.1
SLC30A8* (rs3802177)	0	45 (555)	39.4 (480)	0.003	1	4.74E-03
	1	48.6 (600)	51.4 (626)		1.22 (1.03 - 1.44)	2.31E-02
	2	6.4 (79)	9.3 (113)		1.61 (1.17 - 2.22)	3.63E-03
PPARG (rs1801282)	0	3.2 (39)	1.9 (23)	>0.05	1	>0.1
	1	28 (346)	26.4 (322)		1.54 (0.88 - 2.67)	>0.1
	2	68.8 (849)	71.7 (874)		1.65 (0.96 - 2.84)	0.067
IGF2BP2* (rs4402960)	0	70.6 (871)	65.1 (793)	0.007	1	3.36E-02
	1	26.9 (332)	31.1 (379)		1.22 (1.02 - 1.47)	2.81E-02
	2	2.5 (31)	3.9 (47)		1.49 (0.93 - 2.4)	9.91E-02
CDKAL1 (rs7756992)	0	45.4 (560)	46.5 (567)	>0.05	1	>0.1
	1	43.5 (537)	43.6 (531)		0.96 (0.81 - 1.14)	>0.1
	2	11.1 (137)	9.9 (121)		0.94 (0.71 - 1.24)	>0.1
ADCY5* (rs11717195)	0	16.6 (205)	14.1 (172)	0.021	1	8.75E-02
	1	49.4 (609)	46.8 (570)		1.04 (0.82 - 1.32)	>0.1
	2	34 (420)	39.1 (477)		1.25 (0.97 - 1.6)	8.33E-02
JAZF1 (rs849135)	0	8.5 (105)	7.7 (94)	>0.05	1	>0.1
	1	40.1 (495)	39.9 (486)		1.15 (0.84 - 1.57)	>0.1
	2	51.4 (634)	52.4 (639)		1.2 (0.88 - 1.63)	>0.1
HHEX (rs1111875)	0	37.2 (459)	40.3 (491)	>0.05	1	>0.1
	1	48 (592)	45.2 (551)		0.89 (0.75 - 1.07)	>0.1
	2	14.8 (183)	14.5 (177)		0.92 (0.71 - 1.18)	>0.1
KCNJ11 (rs5219)	0	37.8 (467)	34.2 (417)	>0.05	1	>0.1
	1	45.6 (563)	49.2 (600)		1.18 (0.98 - 1.41)	0.075
	2	16.5 (204)	16.6 (202)		1.11 (0.87 - 1.41)	>0.1
KCNQ1* (rs2237897)	0	17.3 (213)	9.9 (121)	<0.001	1	5.25E-09
	1	44.9 (554)	40.9 (498)		1.53 (1.18 - 1.98)	1.29E-03
	2	37.8 (467)	49.2 (600)		2.17 (1.67 - 2.81)	5.37E-09
TCF7L2* (rs7903146)	0	78.4 (967)	70.4 (858)	<0.001	1	3.98E-04
	1	20.7 (255)	28.5 (347)		1.47 (1.21 - 1.77)	9.43E-05
	2	1 (12)	1.1 (14)		1.51 (0.69 - 3.32)	>0.1
FTO (rs9936385)	0	75.7 (934)	71.6 (873)	0.027	1	>0.1
	1	22.6 (279)	25.5 (311)		1.11 (0.91 - 1.35)	>0.1
	2	1.7 (21)	2.9 (35)		1.64 (0.93 - 2.9)	0.087
CDKN2B* (rs10811661)	0	1.1 (13)	0.5 (6)	0.139	1	9.98E-02
	1	16.8 (207)	15.1 (184)		2.35 (0.86 - 6.45)	9.82E-02
	2	82.2 (1014)	84.4 (1029)		2.64 (0.98 - 7.13)	5.56E-02

a. p-value for frequency distribution was calculated with the Pearson chi-square test, and for the OR with the Wald test.

*SNPs that were statistically significant at p<0.1 in the MLR model. The power (1-β err prob) >0.95 for all SNPs with a p<0.1, except for HNF1A (rs483353044) and IGF2BP2 (rs4402960) which power value were 0.86 and 0.88, respectively.

Supplementary Table S8. Association of 10 SNPs with T2D in a sample of cases and controls of Mexico City stratified by age at T2D diagnosis and gender using MLR models*

Gene (SNP)	Number of Risk Alleles	T2D diagnosed ≤ 45 years old (controls/cases)						T2D diagnosed ≥ 46 years old (controls/cases)					
		Women (759/353)		Men (475/242)		Both genders (1234/595)		Women (759/385)		Men (475/233)		Both genders (1234/618)	
		OR (95% CI)	p	OR (95% CI)	p	OR (95% CI)	p	OR (95% CI)	p	OR (95% CI)	p	OR (95% CI)	p
SLC16A11 (rs75493593)	0	1	2.2E-04	1	1.3E-02	1	6.0E-06	1	p>0.1	1	2.0E-02	1	1.7E-02
	1	1.7 (1.3-2.3)	6.0E-04	1.2 (0.8-1.8)	p>0.1	1.5 (1.2-1.9)	4.2E-04	1.2 (0.9-1.6)	p>0.1	1.6 (1.1-2.3)	1.1E-02	1.4 (1.1-1.7)	5.3E-03
	2	2 (1.4-2.9)	2.5E-04	2 (1.3-3.2)	3.4E-03	2 (1.5-2.6)	2.8E-06	1.2 (0.8-1.7)	p>0.1	1 (0.6-1.6)	p>0.1	1.1 (0.8-1.5)	p>0.1
INS-IGF2 (rs149483638)	0	1	p>0.1	1	6.8E-02	1	5.0E-02	1	p>0.1	1	8.8E-03	1	3.7E-03
	1	1.4 (0.8-2.3)	p>0.1	2.2 (1.1-4.4)	2.5E-02	1.6 (1.1-2.4)	1.8E-02	1.6 (1-2.6)	p>0.1	2.8 (1.3-6.1)	7.7E-03	1.9 (1.3-2.8)	1.9E-03
	2	1.3 (0.8-2.2)	p>0.1	2.2 (1.1-4.3)	2.5E-02	1.6 (1.1-2.4)	1.9E-02	1.5 (1-2.5)	p>0.1	3.2 (1.5-6.9)	2.2E-03	2 (1.3-2.9)	9.4E-04
WFS1 (rs4458523)	0	1	p>0.1	1	6.9E-02	1	7.7E-02	1	p>0.1	1	p>0.1	1	p>0.1
	1	1.4 (0.7-2.7)	p>0.1	2.5 (1-6.3)	6.3E-02	1.6 (1-2.8)	7.6E-02	1.2 (0.7-2.1)	p>0.1	0.9 (0.4-1.9)	p>0.1	1.1 (0.7-1.7)	p>0.1
	2	1.4 (0.8-2.8)	p>0.1	2.9 (1.1-7.3)	2.5E-02	1.8 (1.1-3.1)	2.9E-02	1.1 (0.6-1.9)	p>0.1	1.1 (0.5-2.2)	p>0.1	1 (0.7-1.6)	p>0.1
SLC30A8 (rs3802177)	0	1	6.5E-03	1	1.3E-03	1	1.5E-05	1	2.7E-03	1	p>0.1	1	4.6E-03
	1	1.2 (0.9-1.5)	p>0.1	0.9 (0.6-1.2)	p>0.1	1.1 (0.9-1.3)	p>0.1	1.5 (1.2-1.9)	2.2E-03	1.3 (0.9-1.8)	p>0.1	1.4 (1.1-1.7)	1.8E-03
	2	2.1 (1.3-3.4)	1.5E-03	2.5 (1.4-4.6)	1.9E-03	2.3 (1.6-3.4)	3.9E-06	0.8 (0.4-1.4)	p>0.1	1.2 (0.6-2.4)	p>0.1	1 (0.6-1.5)	p>0.1
IGF2BP2 (rs4402960)	0	1	p>0.1	1	4.1E-03	1	1.5E-02	1	p>0.1	1	p>0.1	1	p>0.1
	1	1.2 (0.9-1.6)	p>0.1	1.7 (1.2-2.5)	3.4E-03	1.3 (1.1-1.7)	1.3E-02	1.1 (0.8-1.4)	p>0.1	1.3 (0.9-1.8)	p>0.1	1.1 (0.9-1.4)	p>0.1
	2	1.3 (0.6-2.6)	p>0.1	2.6 (1-6.8)	5.3E-02	1.7 (1-2.9)	6.8E-02	1.1 (0.5-2.4)	p>0.1	2 (0.7-5.6)	p>0.1	1.3 (0.7-2.4)	p>0.1
ADCY5 (rs11717195)	0	1	p>0.1	1	p>0.1	1	p>0.1	1	p>0.1	1	p>0.1	1	6.5E-02
	1	1.1 (0.7-1.6)	p>0.1	0.8 (0.5-1.4)	p>0.1	1 (0.7-1.3)	p>0.1	1.2 (0.8-1.7)	p>0.1	1.2 (0.7-1.9)	p>0.1	1.1 (0.8-1.5)	p>0.1
	2	1.2 (0.8-1.7)	p>0.1	1 (0.6-1.7)	p>0.1	1.1 (0.8-1.5)	p>0.1	1.4 (1-2.1)	p>0.1	1.5 (0.9-2.5)	p>0.1	1.4 (1-1.9)	3.6E-02
JAZF1 (rs849135)	0	1	p>0.1	1	p>0.1	1	p>0.1	1	p>0.1	1	p>0.1	1	p>0.1
	1	1.2 (0.7-2.1)	p>0.1	1.1 (0.6-2)	p>0.1	1.1 (0.8-1.7)	p>0.1	0.8 (0.5-1.3)	p>0.1	2.1 (1-4.5)	5.2E-02	1.1 (0.8-1.7)	p>0.1
	2	1.2 (0.7-2.1)	p>0.1	1.1 (0.6-2.1)	p>0.1	1.2 (0.8-1.7)	p>0.1	0.9 (0.6-1.4)	p>0.1	2.2 (1.1-4.7)	3.6E-02	1.2 (0.8-1.8)	p>0.1
KCNQ1 (rs2237897)	0	1	1.1E-04	1	1.2E-07	1	9.2E-11	1	3.9E-02	1	2.8E-02	1	2.3E-03
	1	1.7 (1.1-2.7)	1.7E-02	3.5 (1.8-6.6)	1.3E-04	2.2 (1.5-3.1)	3.4E-05	1.5 (1-2.2)	6.6E-02	1 (0.6-1.7)	p>0.1	1.2 (0.9-1.7)	p>0.1
	2	2.5 (1.6-3.9)	6.4E-05	5.9 (3.1-11.2)	5.3E-08	3.3 (2.3-4.8)	1.0E-10	1.7 (1.1-2.5)	1.1E-02	1.6 (1-2.6)	5.1E-02	1.6 (1.2-2.2)	1.7E-03
TCF7L2 (rs7903146)	0	1	1.3E-02	1	3.4E-04	1	5.7E-06	1	p>0.1	1	4.4E-03	1	3.6E-02
	1	1.6 (1.2-2.1)	3.7E-03	2 (1.3-2.9)	5.2E-04	1.7 (1.4-2.2)	6.4E-06	1.0 (0.8-1.4)	p>0.1	1.9 (1.3-2.8)	9.8E-04	1.3 (1-1.6)	2.9E-02
	2	1.5 (0.4-5.1)	p>0.1	4.6 (1.2-17.1)	2.2E-02	2.7 (1.2-6.3)	2.1E-02	0.4 (0.1-2)	p>0.1	0.6 (0.1-5.4)	p>0.1	0.4 (0.1-1.7)	p>0.1
CDKN2B (rs10811661)	0	1	2.1E-02	1	p>0.1	1	8.3E-02	1	p>0.1		p>0.1	1	p>0.1
	1	4.1 (0.5-33.5)	p>0.1	0.9 (0.1-8.3)	p>0.1	2.3 (0.6-8.6)	p>0.1	1.84 (0.5-7)	p>0.1	ND	p>0.1	2.2 (0.6-8.2)	p>0.1
	2	6.4 (0.8-50.4)	8.0E-02	0.9 (0.1-7.6)	p>0.1	2.9 (0.8-10.6)	1.0E-01	1.91 (0.5-7.1)	p>0.1	ND	p>0.1	2.2 (0.6-8.1)	p>0.1

p-values were calculated with the Wald test. ND = ORs were not calculated because no one men with T2D diagnosed ≥ 46 years has the homocigote genotype for the alternative allele.
*The power (1-β err prob) >0.95 for all SNPs with a p<0.1.

Supplementary Table S9. Contribution of BMI, waist, PH-T2D, and 16 SNPs in T2D variability stratified by gender using MLR models in the cross-sectional study*

Variable	Block	Women (69/132)				Men (30/61)				Both genders (99/193)			
		-2 log LH	r^2	pb	pm	-2 log LH	r^2	pb	pm	-2 log LH	r^2	pb	pm
SNPs	1	254	0.03	0.084	0.084	93	0.31	0.001	0.001	359	0.07	0.023	0.023
PH	2	248	0.07	0.075	0.038	93	0.31	0.74	0.001	355	0.09	0.093	0.013
BMI	3	230	0.19	<0.0001	<0.0001	84	0.4	0.004	<0.0001	326	0.21	<0.0001	<0.0001
Waist	4	230	0.19	0.24	<0.0001	84	0.4	0.43	<0.0001	323	0.22	0.074	<0.0001

r^2 (Nagelkerke) and p-values (omnibus test) were calculated with the multivariate logistic regression models.

LH= likelihood, PH=parental history of T2D; BMI= raw body mass index; pb= p-value of the block; pm= p-value of the model.

* In parentheses is the number of cases/controls analysed in each group. The power (1-β err prob) was calculated only for "Both genders" multivariate logistic regression (MLR) model. It was > 0.95 for BMI, waist and PH, and two of the three genes with a p<0.1 (JAZF1, FTO) and 0.71 for KCNQ1. The number of cases of males and females is very small, well below the N recommended for MLR studies (50-100).

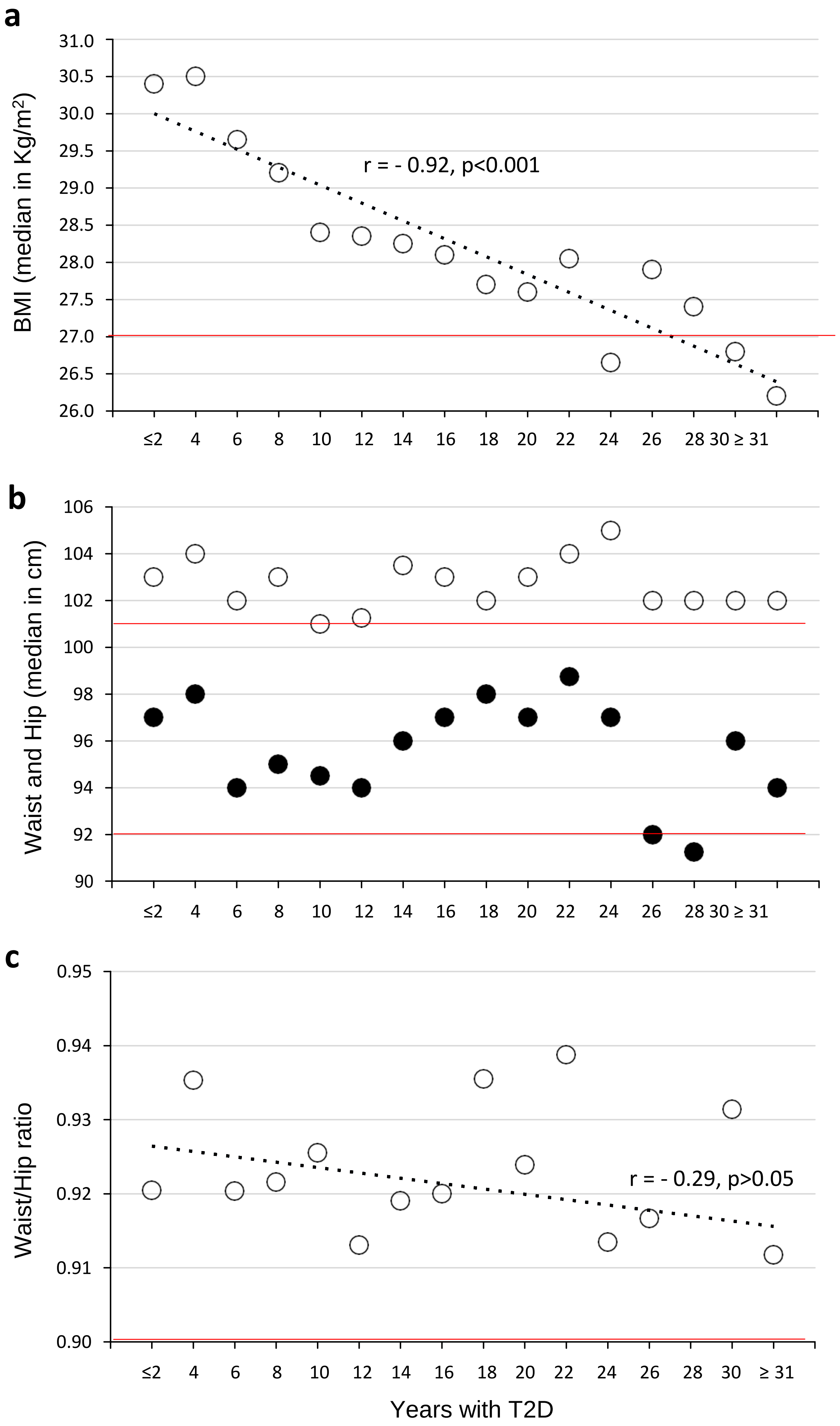

a
BMI (median in Kg/m²)
31.0
30.5
30.0
29.5
29.0
28.5
28.0
27.5
27.0
26.5
26.0
r = - 0.92, p<0.001
≤2
4
6
8
10
12
14
16
18
20
22
24
26
28 30 ≥ 31
b
Waist and Hip (median in cm)
106
104
102
100
98
96
94
92
90
≤2
4
6
8
10
12
14
16
18
20
22
24
26
28 30 ≥ 31
c
Waist/Hip ratio
0.95
0.94
0.93
0.92
0.91
0.90
r = - 0.29, p>0.05
≤2
4
6
8
10
12
14
16
18
20
22
24
26
28
30
≥ 31
Years with T2D

Supplementary Fig. 1. Trend of body mass index (BMI), circumferences of waist (W) and hip (H) and WH ratio (WHR) over the years having type 2 diabetes in the clinical replica case-control study. The red line indicates the median value of the control non-diabetic group. The correlation coefficient was calculated using the Pearson correlation test.

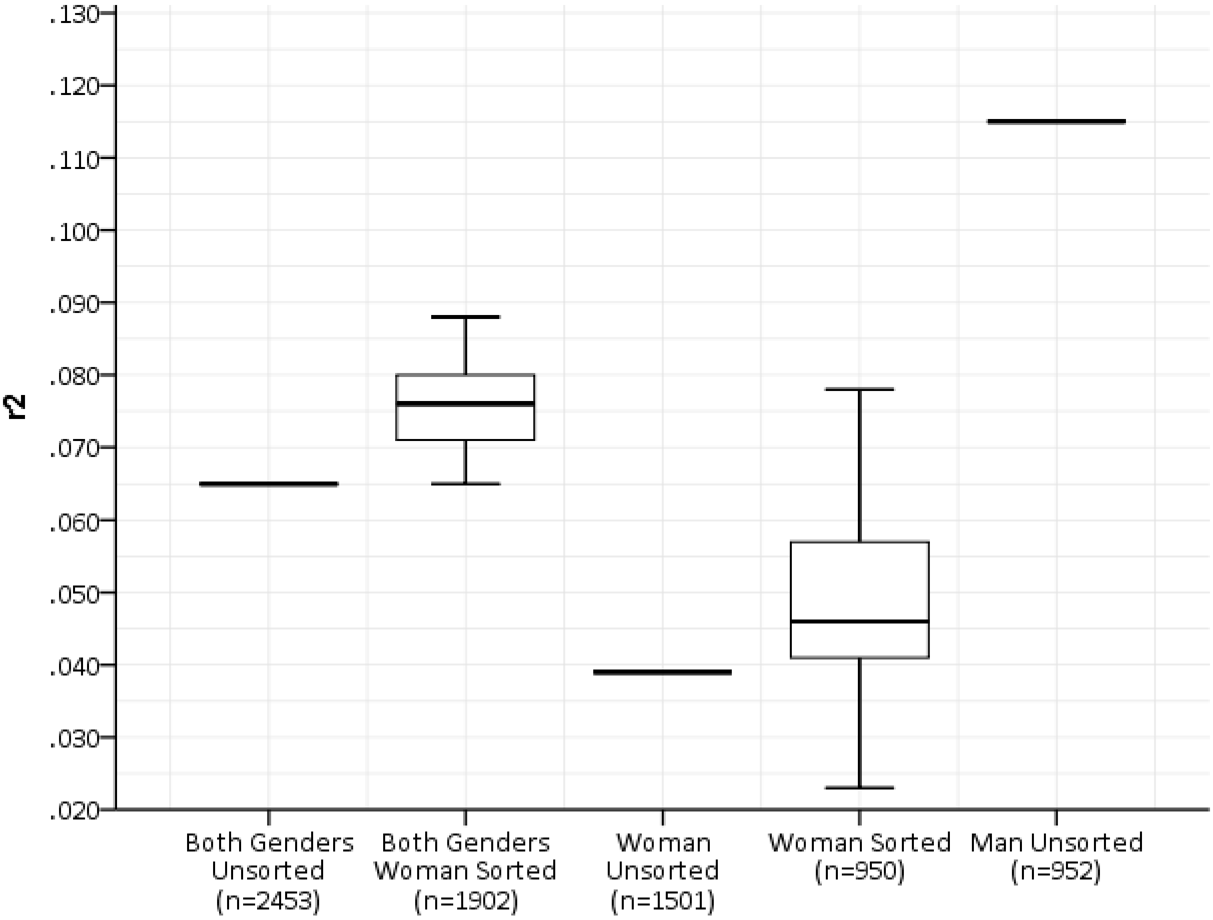

Supplementary Fig. 2. Influence (r^2) of genes in T2D. To include a number of women that was equal to the number of men and to avoid a bias, 100 different groups of 950 women were randomly selected from a total of the 1501 women included in the main case-control study. For each new group of women and both sexes together (n=1902), the r^2 value for the 16 SNPs explored was calculated using the MLR model. Box plot graphs show the distribution of the data for the 100 newly constructed groups. The upper and lower boundaries of the boxes represent the 75th and 25th percentiles, respectively. The black line within the box represents the median value, and the whiskers represent the minimum and maximum values that lie within 1.5x of the interquartile range from the end of the box. Where there is only one line, it indicates the value found when all of the samples included in the study were entered into the MLR model, men (n=952), women (n=1501) or both (n=2453).
